# Identification of *Datura innoxia* as a potential source of antimycobacterial components

**DOI:** 10.3389/fmicb.2025.1553282

**Published:** 2025-07-01

**Authors:** Sajjad Ahmed Khan, Muzafar Ahmad Rather, Muhammad Sheeraz Ahmad, Ziyi Jia, Anthony D. Baughn, Sajid Iqbal, Syed Mehmood Qadir, Sabira Tahseen, Muhammad Umer Khan, Peter W. Villalta, W. Thomas Shier

**Affiliations:** ^1^University Institute of Biochemistry and Biotechnology, PMAS-Arid Agriculture University Rawalpindi, Rawalpindi, Pakistan; ^2^National Reference Laboratory for Tuberculosis, National TB Control Program, Islamabad, Pakistan; ^3^Department of Medicinal Chemistry, College of Pharmacy, University of Minnesota, Minneapolis, MN, United States; ^4^Department of Microbiology & Immunology, University of Minnesota Medical School, Minneapolis, MN, United States; ^5^Department of Microbiology, Quaid-i-Azam University, Islamabad, Pakistan; ^6^Institute of Molecular Biology and Biotechnology, The University of Lahore, Lahore, Pakistan; ^7^Masonic Cancer Center, University of Minnesota, Minneapolis, MN, United States

**Keywords:** *Mycobacterium tuberculosis*, *Datura innoxia*, antitubercular activity, LC-MS/MS, scopolamine, milbemycin A3 oxime

## Abstract

*Datura innoxia* is a medicinal plant from the Solanaceae family, having medicinal properties and some toxic effects. It is widely distributed across Asia, Africa, Europe, the Americas, and other tropical and subtropical regions, where it is utilized by local pharmaceutical industries. In this study, bioassay-guided fractionation and LC-MS/MS analysis were used for the identification of secondary metabolites with anti-tuberculosis activity in methanolic leaf extracts of *D. innoxia*. Bioassay-guided fractionation was conducted using normal and reverse phase column chromatography, and the fractions were assayed for antituberculosis activity *in vitro* by serial dilution in *Mycobacterium tuberculosis* H37Ra cultures. The structures of known secondary metabolites in the purified extracts were identified using LC-ESI-MS/MS mass spectroscopy. A purified fraction of the methanolic extract of *D. innoxia* leaves inhibited *M. tuberculosis* growth at concentrations as low as 25 μg/mL. Metabolic profiling with LC-ESI-MS/MS enabled the identification of the purified extract of 16 known metabolites, including loliolide, scopolamine, kuromanin, isoquercitrin, moupinamide, methyl isoquinoline-3-carboxylate, trans-3-Indoleacrylic acid, tyramine, (3β,5ξ,9ξ)-3,6,19-trihydroxyurs-12-en-28-oic acid, milbemycin A3 oxime, methyl jasmonate, nicotinamide, methyl ferulate, trifolin, 2-[(1S,2S,4aR,8aS)-1-hydroxy-4a-methyl-8-methylidene-decahydronaphthalen-2-yl]prop-2-enoic acid, and methyl 4-hydroxycinnamate. These results indicate that *D. innoxia* is a rich natural source of potential antitubercular secondary metabolites.

## 1 Introduction

*Mycobacterium tuberculosis* (Mtb) is a Bacillus bacterium that causes the infectious disease tuberculosis (TB). TB ranks among the top 10 diseases in the world in terms of both mortality and morbidity (Rodriguez-Takeuchi et al., 2019). According to the WHO global report, an estimated 10.6 million individuals have active *Mtb* infections, resulting in approximately 1.30 million reported deaths (Chunrong et al., 2023), whereas approximately one-fourth of the world population (≈ 2 billion people) is estimated to be latently infected with *Mtb* (Chin et al., 2023). Despite significant treatment advances, TB remains a serious global health concern (Sharifi-Rad et al., 2020). Approximately 40 years ago, a standardized 6-month treatment regimen for tuberculosis was established, based on the use of four first-line drugs: isoniazid, rifampicin, ethambutol, and pyrazinamide. This regimen is widely recommended and has been shown to cure approximately 85% of patients with drug-sensitive tuberculosis (WHO, 2013). One of the biggest obstacles to TB management worldwide is the rapid spread of drug-resistant TB strains. These strains are currently present in most nations and are growing alarmingly. Multidrug-resistant (MDR) TB isolates that are resistant to isoniazid and rifampicin, the two first-line medications for TB therapy, have been found in every country surveyed (Cazzaniga et al., 2021).

Natural products have played a vital role in the discovery of new drugs; today, more than 25% of conventional drugs on the market are either directly or indirectly derived from plant secondary metabolites (Marealle et al., 2023). Similarly, medicinal plants and their extracts have served as valuable resources for the discovery and development of alternative treatments for TB (Mpeirwe et al., 2023; Tuyiringire et al., 2020; Karimi, 2023). According to floral research, there are approximately 500,000 plant species on the planet, and 120,000 of those species have biologically active compounds that can be used to treat illnesses (Kallassy, 2017; Houghton, 2001), particularly in developing countries, where the World Health Organization estimated that 70%−80% of the population depends on traditional medicines for their primary source of medication (Akinyemi et al., 2005; Maluleka and Ngoepe, 2018).

Datura is a genus of medicinal herbs in the nightshade family (Solanaceae), commonly known as jimsonweed or thornapple, which have both toxic and medicinal properties (Sharma et al., 2021). Datura species are widely cultivated in Asia, Africa, Europe, America, and other tropical and subtropical regions for use in herbal medicine preparations (Gaire and Subedi, 2013; Lakusic et al., 2017). Datura species have been reported to possess antidiabetic, antimicrobial, anti-cancer, anti-asthmatic, anti-inflammatory, analgesic, antioxidant, cytotoxic, insecticidal, and neurological activities, and wound healing (Alam et al., 2021; Al-Snafi, 2017).

## 2 Materials and methods

### 2.1 Plant collection and identification

**Figure 1:**
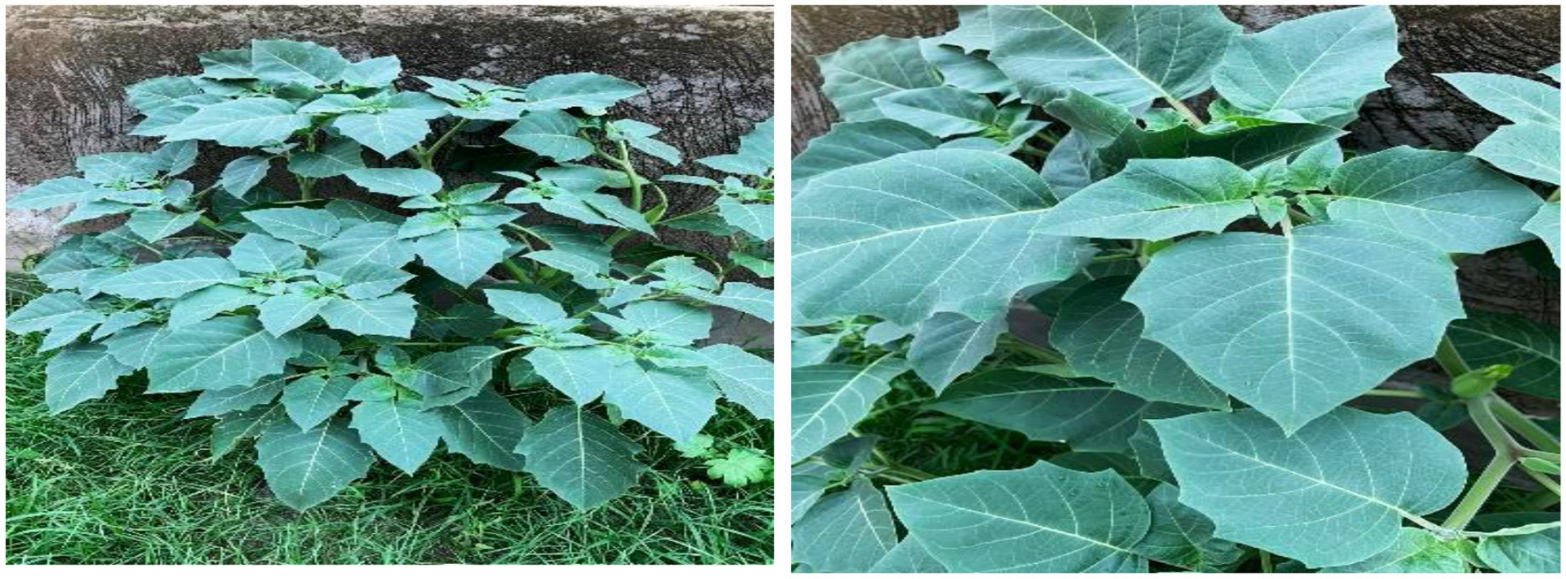
*Datura innoxia* whole plant **(left)** and leaves **(right)**.

Leaves of *Datura innoxia* (Figure 1) were collected between March and June 2020, near Islamabad, Pakistan. The plant material was identified and authenticated by Professor Rahmatullah Quraishi, Department of Botany, PMAS Arid Agriculture University, Rawalpindi, Pakistan. A voucher specimen was submitted to the Herbarium of Medicinal Plants and assigned a unique herbarium number (PMAS-177).

### 2.2 Extraction of plant material

The leaves of *D. innoxia* were washed with tap water to eliminate impurities and dried at room temperature. The dried leaves were crushed into a powder. To obtain crude extracts of the dried powder, successive extraction with maceration was conducted as previously used (Ahmed et al., 2023). Briefly, powdered plant material (200 g) was suspended in 1,000 mL of methanol (plant biomass to solvent ratio of 1:5 w/v) in an Erlenmeyer flask and shaken for 48 h at room temperature. The initial filtration was performed using a muslin cloth, followed by fine filtration with Whatman No. 1 filter paper. To increase the extract yield, an additional aliquot of methanol (at a plant biomass to solvent ratio of 1:3 w/v) was added to the extraction residue, which was then subjected to an additional 72 h extraction and filtered. The combined filtrates were evaporated using a rotary evaporator at lower pressure to yield a crude methanolic extract (CME). This extract was subsequently fractionated into the three samples: non-polar fraction (CME extracted with n-hexane), moderately polar fraction (residue from n-hexane extraction then extracted with ethyl acetate), and highly polar fraction (residue from ethyl acetate extraction then extracted with distilled water). A rotary evaporator was used to evaporate the solvents at reduced pressure, and all fractions were tested for anti-mycobacterial activity. The most active crude fraction (ethyl acetate) was stored at 2–4°C for further analysis (Ahmed et al., 2023; Jabeen et al., 2022). The *D. innoxia* extracts and their fractions demonstrated sufficient stability, with no detectable changes in anti-TB activity observed in repeated experiments.

### 2.3 Fractionation of ethyl acetate extract

Normal-phase column chromatography was used to fractionate 6.30 g of ethyl acetate extract using silica gel (60–120 mesh) (Ahmed et al., 2023). The fractionation was carried out using a stepwise gradient of n-hexane:ethyl acetate in the following volume ratios: 100:0, 80:20, 60:40, 40:60, 20:80, and 0:100 (v/v). This was followed by elution with ethyl acetate:methanol in ratios of 100:0, 80:20, 60:40, 40:60, 20:80, and 0:100 (v/v). The collected fractions were evaporated under reduced pressure. The yield (%) of each fraction was calculated using the following formula:


$$\begin{array}{l} Yield\;\left(\%\right)=\frac{net\;weight\;of\;fraction}{total\;weight\;of\;crude\;ethyl\;acetate\;extract}\;x\;100 \end{array}$$

### 2.4 Bacterial culture conditions

*Mycobacterium tuberculosis* H37Ra was grown in Middlebrook 7H9 broth medium supplemented with 0.2% (v/v) glycerol (Sigma Chemical Co.), 10% (w/v) oleic acid, albumin, dextrose, catalase (OADC; Difco), and 0.05% (w/v) tyloxapol (Sigma). Minimum bactericidal concentration (MBC) measurements were performed on Middlebrook 7H10 agar media supplemented with 0.2% glycerol and 10% (w/v) OADC (Martin et al., 2005).

### 2.5 Minimum inhibitory concentration and minimum bactericidal concentration determinations

3-[4,5-Dimethylthiazol-2-yl]-2,5-diphenyltetrazolium bromide (MTT) assay was used to determine the MICs of the crude plant extracts, with minor modifications to the method described by Martin et al. (2005). Briefly, the fractions were evaporated to dryness, and the residues were accurately weighed before being dissolved in DMSO to prepare stock solutions of known concentrations. Two-fold serial dilutions of the samples were prepared to achieve final concentrations ranging from 200 μg/mL to 12.5 μg/mL. Subsequently, 5 μL of the diluted samples was added to a 96-well plate, followed by 95 μL of the H37Ra culture (final OD_600_ = 0.01). The cultures were incubated for 7 days at 37°C, and MICs were recorded by visual observations. Each concentration above the visually observed MICs was serially diluted, and 10 μL of each dilution was plated on Middlebrook 7H10 agar plates. The agar plates were incubated at 37°C for 3 weeks. The MBC was recorded as the lowest concentration that resulted in a 99% reduction in colony-forming units (CFUs) in the initial inoculum. MTT solution (10 μL of 5 mg/mL) was added to all the wells of the 96-well plate, followed by overnight incubation. Then, 50 μL of formazan solubilization buffer (Abate et al., 1998) was added, and incubation was continued for at least 4 h at 37°C. A color change from yellow to violet indicated bacterial growth, and MICs were interpreted accordingly (Vilchèze et al., 2011).

### 2.6 Preparative thin layer chromatography

Preparative TLC was carried out on the most active fraction (F10) of the crude extract of *D. innoxia* leaves (ethyl acetate) on 250-micron silica gel layers developed with chloroform: ethanol (75:25). Distinct bands were individually scraped from the plate, transferred to mini-columns, and eluted with methanol. The eluate was filtered through a paper and concentrated under reduced pressure (Nimbeshaho et al., 2020).

### 2.7 Reverse phase column chromatography

Sample material eluted from the prep-TLC band (F10B5), measuring 4 mg, was dissolved in the minimum amount of MeOH and applied to the column with 5 g of octadecyl-functionalized silica gel to collect the effluent (Choudhari et al., 2009). Columns were eluted sequentially with 10 mL of the following percentages of MeOH in water: 10%, 20%, 30%, 40%, 50%, 60%, 70%, 80%, 90%, and 100% MeOH, and the effluents were collected as fractions. The collected fractions were evaporated under reduced pressure, and the residues were assayed for antituberculosis activity using the MTT assay described above.

### 2.8 LC-ESI-MS/MS data acquisition and analysis

LC-MS/MS phytochemical profiling of the extracted fraction RC08 was carried out using an Orbitrap Fusion mass spectrometer (Thermo Scientific, Waltham, MA, USA) connected to a Dionex UltiMate 3000 RSLCnano UPLC system. The sample (5 μl) injections were subjected to chromatographic separations using a mobile phase of water with 0.1% formic acid (A) and acetonitrile (B) on an Acquity HSS (Waters, Milford, MA) C18 reversed-phase column (100 × 2.1 mm, 1.8 μm particle size). The initial conditions were 2% B for 3 min, followed by a linear gradient to 95% B over 49 min (long) and 12 min (short) with a 2-min hold at 95% B. The column was then re-equilibrated with 2% B for the next run. Electrospray ionization was used to acquire MS data, with full scan Orbitrap detection (m/z 100–1,000, resolution 120,000) and data-dependent HCD fragmentation (stepped 20%, 35%, and 60%) with a 1-s cycle time, 6 s dynamic exclusion, 1.6 Da quadrupole isolation width, exclusion mass width ±10 ppm, and 15,000 resolution Orbitrap detection. Each sample was performed independently in the negative (M – H^+^) and positive (M + H^+^) modes. Compound Discoverer 3.3 (Thermo Scientific, Waltham, MA) was used for data processing, and analyte identification was done by searching the Thermo Scientific mzCloud and NIST 2020 high-resolution mass spectral databases (Flamini, 2013).

## 3 Results

### 3.1 Bioassay-guided fractionation of the *D. innoxia* leaf ethyl acetate extract

**Figure 2:**
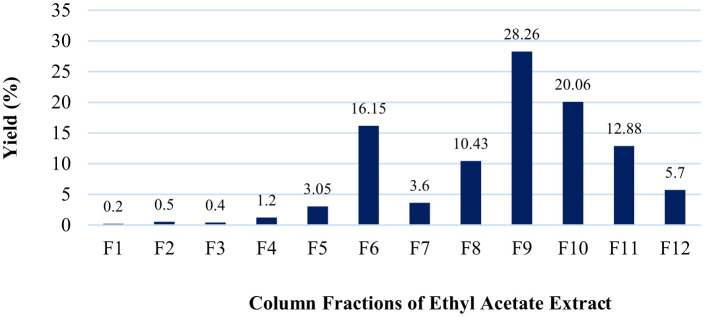
Fractionation yield (%) was obtained from the ethyl acetate extract of the *D. innoxia leaves*.

A total of 12 fractions were obtained from the column chromatography of the ethyl acetate extract of *Datura innoxia* leaves. The highest yield was obtained for fraction #F9 (28.26%), followed by F10 (20.06%), F6 (16.15%), and others (Figure 2).

Fractions of extracts with a yield ≥1.23% (77.5 mg) were assayed for antitubercular activity in cultures of *M. tuberculosis* H37Ra over a range of concentrations (200 μg/mL to 3.15 μg/mL). Fractions F05, F06, F10, and F12 significantly inhibited Mtb growth at 200 μg/mL (Table 1).

**Table 1:** Antitubercular activity of fractions obtained using normal phase column chromatography.

**S. no**	**Fractions of ethyl acetate extract**	**Antitubercular activity against *M. tuberculosis* H37Ra**
		**MICs (**μ**g/mL)**
1	F04	>200
2	F05	200
3	F06	200
4	F07	>200
5	F08	>200
6	F09	>200
7	**F10** ^*^	**200**
8	F11	>200
9	F12	200

* F10 was selected for further analysis due to its high yield. Bold values represent the most active fractions, showing the lowest MIC and MBC values against *Mtb*.

#### 3.1.1 Preparative TLC fractionation

Fraction F10, which had the highest total activity, was further purified by preparative TLC, yielding 11 distant bands under UV visualization (Supplementary Figure 15). All bands were individually scraped from the plates, extracted with methanol, the solutions were evaporated, and the residues were assayed for antitubercular activity in *M. tuberculosis* H37Ra cultures. Fraction F10B5 exhibited the highest antitubercular activity (MIC = 25 μg/mL; MBC = 100 μg/mL) (Table 2).

**Table 2:** Antitubercular activity of PTLC fractions from fraction #10.

**Antitubercular activity against** ***M. tuberculosis*** **H37Ra**		
**Fraction**	**MIC (**μ**g/mL)**	**MBC (**μ**g/mL)**
F10B1	>200	>200
F10B2	>200	>200
F10B3	200	>200
F10B4	50	>200
**F10B5**	**25**	**100**
F10B6	100	200
F10B7	200	200
F10B8	200	>200
F10B9	50	>200
F10B10	50	>200
F10B11	50	100

Bold values represent the most active fractions, showing the lowest MIC and MBC values against *Mtb*.

#### 3.1.2 Reverse-phase column fractionation

Fraction F10B5 was purified on a reverse-phase column, resulting in 10 sub-fractions, which were assayed for antitubercular activity. Fraction RC08 exhibited the most active MIC and MBC values at 50 μg/mL and 100 μg/mL, respectively (Table 3). The lower specific activity of RC08 relative to F10B5 may represent assay variability or removal of a co-activator or other active components.

**Table 3:** MICs and MBCs of RC fractions from fraction F10B5.

**Reverse phase column fractionations**	**Antitubercular activity against** ***M. tuberculosis*** **H37Ra**	
	**MIC (**μ**g/mL)**	**MBC (**μ**g/mL)**
RC01	>200	>200
RC02	>200	>200
RC03	>200	>200
RC04	>200	>200
RC05	>200	>200
RC06	>200	>200
RC07	>200	>200
**RC08**	**50**	**100**
RC09	100	100
RC10	100	200

Bold values represent the most active fractions, showing the lowest MIC and MBC values against *Mtb*.

### 3.2 Characterization and metabolomic profiling of the RC08 fraction of *D. innoxia* leaf extract

**Figure 3:**
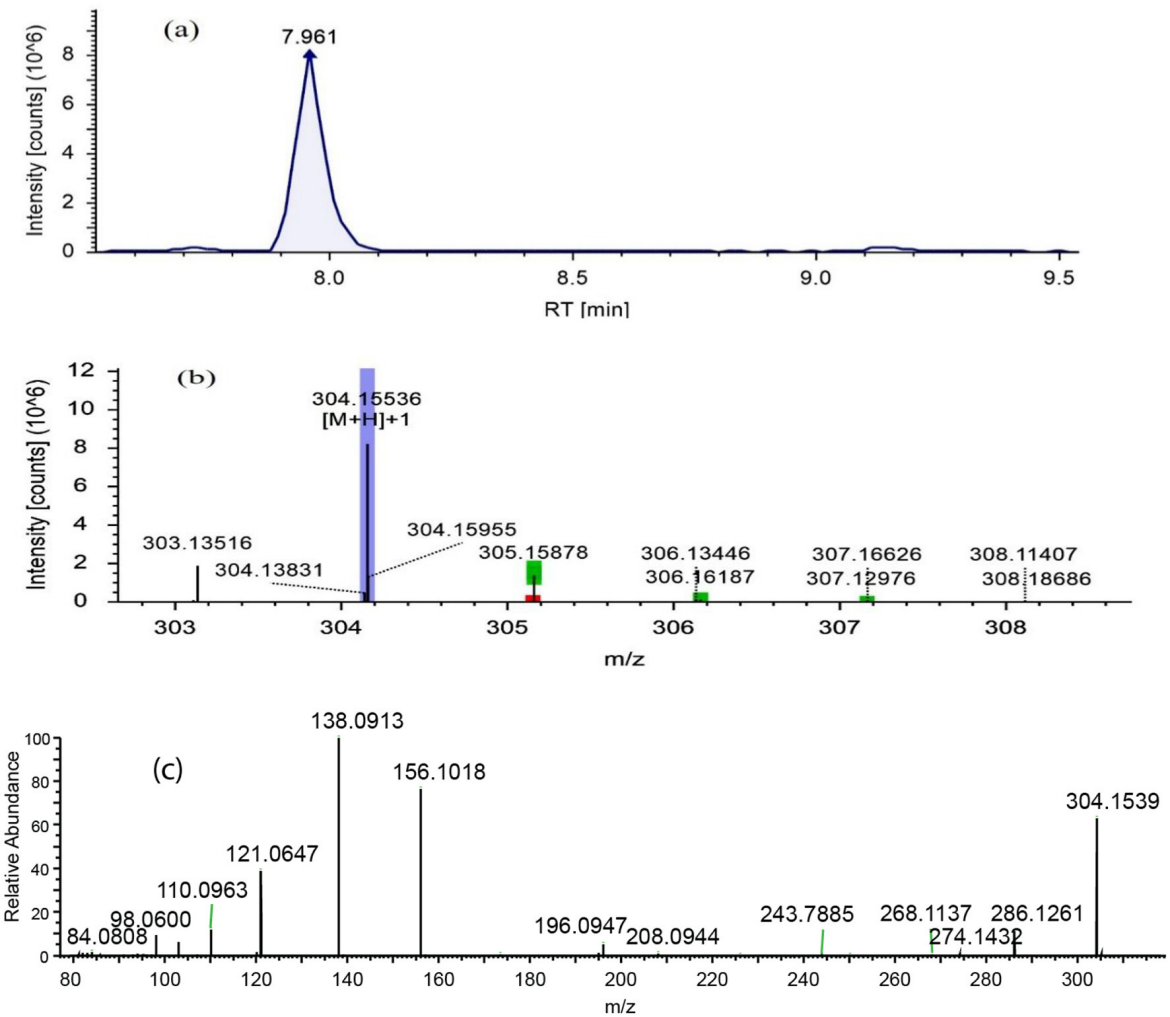
LC-ESI-MS/MS chromatograms of fraction RC08 in positive ion mode were used for the detection of scopolamine. **(a)** Extracted ion chromatogram, **(b)** high-resolution mass spectrum (MS1), and **(c)** fragmentation mass spectrum for the mass ion at m/z 304.15536 (MS2). RT, retention time.

**Figure 4:**
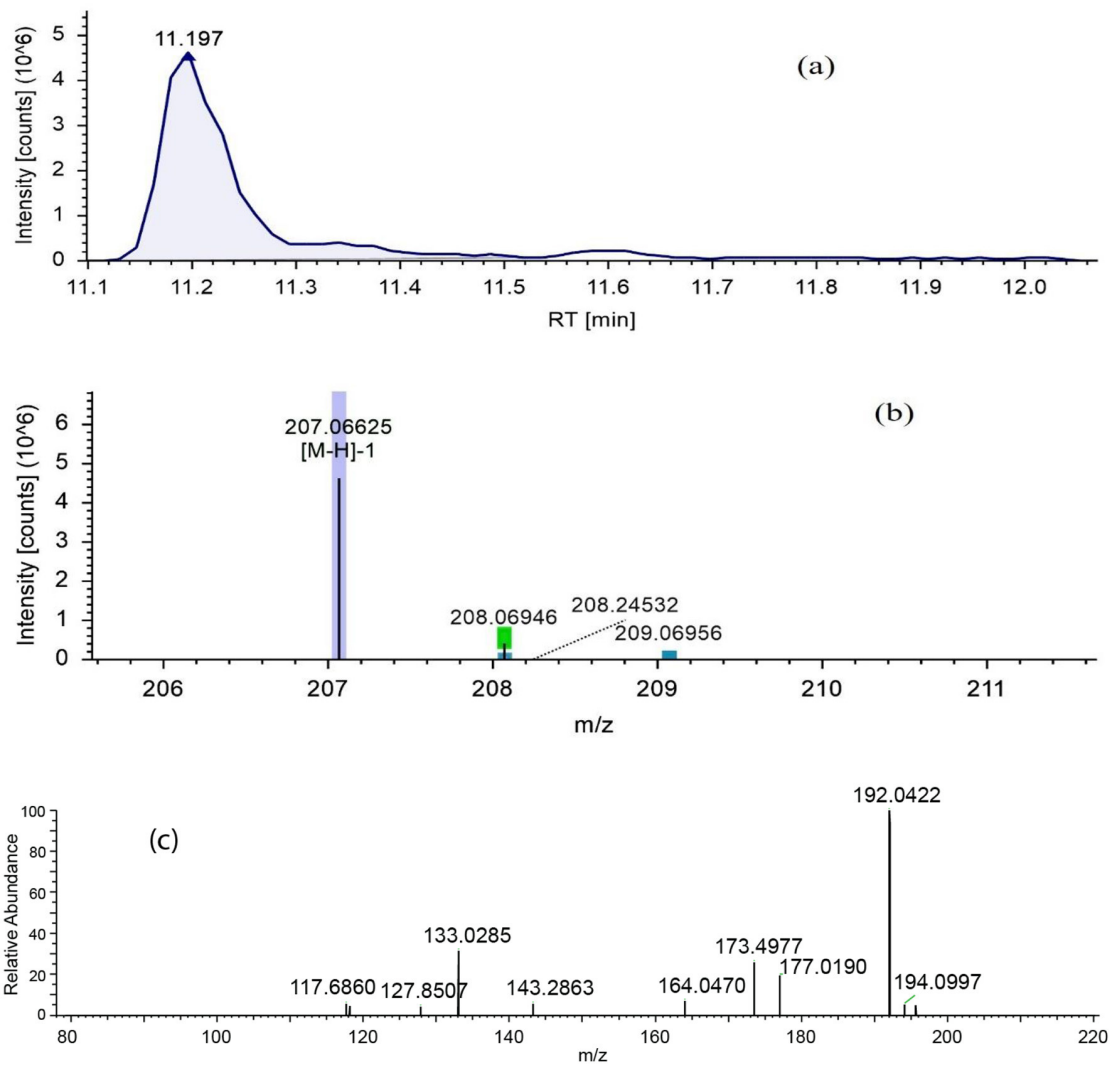
LC-ESI-MS/MS chromatograms of fraction RC08 in negative ion mode for the detection of methyl ferulate. **(a)** Extracted ion chromatogram, **(b)** high resolution mass spectrum (MS1), and **(c)** fragmentation mass spectrum for the mass ion at m/z 207.06625 (MS2). RT, retention time.

**Figure 5:**
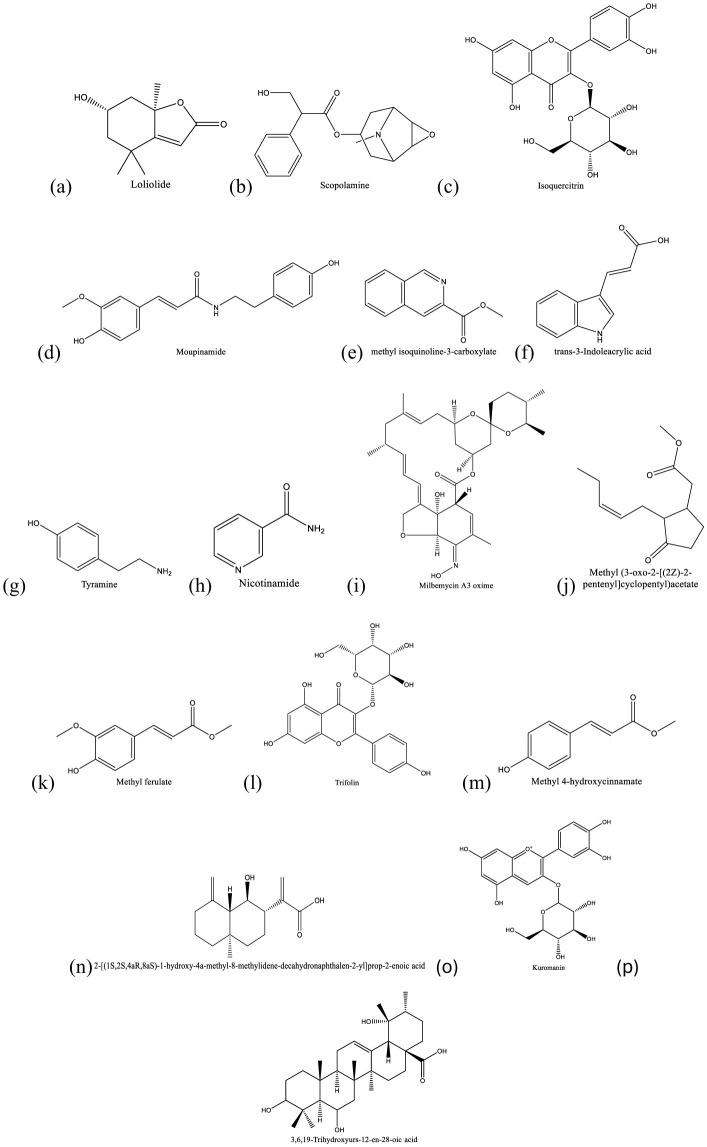
Structures of secondary metabolites identified by LC-ESI-MS/MS. **(a)** Loliolide, **(b)** scopolamine, **(c)** isoquercitrin, **(d)** moupinamide, **(e)** methyl isoquinoline-3-carboxylate, **(f)** trans-3-Indoleacrylic acid, **(g)** tyramine, **(h)** nicotinamide **(i)** milbemycin A3 oxime, **(j)** methyl jasmonate, **(k)** methyl ferulate, **(l)** trifolin, **(m)** methyl 4-hydroxycinnamate and **(n)** 2-[(1S,2S,4aR,8aS)-1-hydroxy-4a-methyl-8-methylidene-decahydronaphthalen-2-yl]prop-2-enoic acid, **(o)** kuromanin, and **(p)** (3β,5ξ,9ξ)-3,6,19-trihydroxyurs-12-en-28-oic acid.

Fraction RC08 was subjected to metabolic profiling using LC-MS/MS. The reverse-phase LC column and gradient conditions used in LC-MS/MS resulted in much better separation and resolution than the step gradient open column used in the previous fractionation step. An example of a chromatogram obtained in positive ion mode is given in Figure 3, and one in negative ion mode is given in Figure 4. Additional chromatograms are provided in Supplementary Figures 1–14. The data analysis in this study identified 16 known compounds, including 12 compounds in the positive ion mode spectrum (Table 4) and four compounds in the negative ion spectrum (Table 5) based on the high-resolution mass spectrum (which identifies the molecular weight and elemental composition) and the electron impact spectra by comparison with available databases. The structures of the identified metabolites are given in Figure 5.

**Table 4:** Secondary metabolites identified in the RC08 fraction of *Datura innoxia leaves* by positive ion mode LC-MS/MS analysis.

**Metabolites identified**	**Retention time (min)**	**Chemical formula**	**Molecular weight**	**[M+H]^+1^ m/z**
Loliolide	9.63	C_11_H_16_O_3_	196.1105	197.1177
Scopolamine	7.96	C_17_H_21_NO_4_	303.1480	304.1552
Kuromanin	8.96	C_21_H_20_O_11_	448.1014	449.1086
Isoquercitrin	9.34	C_22_H_21_O_11_	462.1177	463.1249
Moupinamide	8.65	C_18_H_19_NO_4_	313.1325	314.1397
methyl isoquinoline-3-carboxylate	11.22	C_11_H_9_NO_2_	187.0636	188.0708
trans-3-Indoleacrylic acid	7.47	C_11_H_9_NO_2_	187.0641	188.0713
Tyramine	1.13	C_8_H_11_NO	137.0845	138.0917
(3β,5ξ,9ξ)-3,6,19-Trihydroxyurs-12-en-28-oic acid	10.38	C_30_H_48_O_5_	488.3521	489.3593
Milbemycin A3 oxime	11.27	C_31_H_43_NO_7_	541.3051	542.3123
Methyl jasmonate	12.19	C_13_H_20_O_3_	224.1418	225.149
Nicotinamide	1.81	C_6_H_6_N_2_O	122.0485	123.0557

**Table 5:** Secondary metabolites identified in the RC08 fraction of *Datura innoxia leaves* by negative ion mode LC-MS/MS analysis.

**Metabolites identified**	**Retention time (min)**	**Chemical formula**	**Molecular weight**	**[M-H^+^]^−1^ m/z**
Methyl ferulate	11.19	C_11_H_12_O_4_	208.0735	207.0663
Trifolin	8.95	C_21_H_20_O_11_	448.1008	447.0936
2-[(1S,2S,4aR,8aS)-1-hydroxy-4a-methyl-8-methylidene-decahydronaphthalen-2-yl]prop-2-enoic acid	11.51	C_15_H_22_O_3_	250.1569	249.1497
Methyl 4-hydroxycinnamate	11.01	C_10_H_10_O_3_	178.0629	177.0557

## 4 Discussion

Natural products have a proven record for the development of new drugs, including potential anti-TB agents (Oosthuizen et al., 2019). In the present research, Datura species were screened for antimycobacterial potential in cultures of *Mycobacterium tuberculosis* H37Ra (Vilchèze et al., 2011). Numerous secondary metabolites with pharmaceutical potential have been found in Datura species (Alam et al., 2021; Al-Snafi, 2017). The most active of the Datura species extracts included in the study, that of *D. innoxia*, was subjected to bioassay-guided fractionation using solvent extraction and chromatographic techniques to reduce the number of inactive components and to reduce the potential for masking biological activity with impurities (Sytar and Smetanska, 2023).

Among the known compounds identified as secondary metabolites in *D. innoxia* was loliolide, a monoterpene lactone and benzofuran found in many plants, which exhibits various biological activities, including antifungal, antitumor, cytoprotective, antibiotic, antioxidant, antimalarial, and anticancer properties (Silva et al., 2021; Yang et al., 2011; Grabarczyk et al., 2015). Scopolamine is a tropane alkaloid belonging to the Solanaceae family of plants, including angel's trumpet, devil's trumpet, henbane, mandrake, deadly nightshade, and corkwood (Isopencu et al., 2023). Scopolamine, first approved by the U.S. Food and Drug Administration in 1979, is used to prevent motion sickness and postoperative nausea, acting by an anticholinergic mechanism (Swaminathan et al., 2020; Palazón et al., 2008). Moupinamide (N-trans-feruloyltyramine), which has been found in a variety of plants, including eggplant (Song et al., 2021), has some potential therapeutic activities, including inhibition of COX 1 and COX 2 (Park, 2009), stimulation of lipophagy by dihydroceramides (Lee et al., 2021) making it a possible non-alcoholic fatty liver disease therapeutic and cytotoxicity with SW480 cells (Villada Ramos et al., 2023). Kaempferol 3-O-galactoside (trifolin), a member of the flavonol group, has been extracted from medicinal plants and reported to have anticancer effects against promyelocytic leukemia, histiocytic lymphoma, skin melanoma, and lung cancer (Imran et al., 2019). Tyramine has peripheral cardiovascular effects when orally ingested, making it potentially useful for treating hypotension (Blob et al., 2007). Methyl 4-hydroxycinnamate is found in a variety of plants and has potential therapeutic applications as a melanin synthesis inhibitor, anti-inflammatory agent, and antifungal agent (Roulier et al., 2020). Trans-3-indoleacrylic acid is found in a wide variety of plant sources, such as canola straw, and is of interest as an algaecide (Effiong et al., 2022). Trans-3-indoleacrylic acid is also produced by gut bacteria, which facilitates the development of colorectal cancer (Cui et al., 2024). Other metabolites identified in the *D. innoxia* extract were primary plant metabolites, including nicotinamide, methyl jasmonate, and methyl ferulate. Primary plant metabolites that co-purify with the antitubercular activity in *D. innoxia* extract would be expected to be detected and identified.

The only secondary metabolite identified in the *D. innoxia* extract with reported antitubercular activity is milbemycin oxime (Muñoz-Muñoz et al., 2021). Milbemycin oxime has been reported to be more active against *M. tuberculosis* and other *Mycobacterium* species than other milbemycins or closely related avermectins, with an MIC lower than 8 μg/mL (Lim et al., 2013). Milbemycin oxime is produced by *Streptomyces hygroscopicus* subspecies *aureolacrimosus* (Takiguchi et al., 1983) and has not been reported to be produced by *D. innoxia*. Examination of the total ion flow in the chromatogram at the milbemycin oxime peak indicated that it was present only in trace amounts in the *D. innoxia* extracts. Assessment of the antitubercular activity of a series of pure, commercially available authentic standards for components identified in the *D. innoxia* extract and their amounts in the various purification fractions indicated that antitubercular activity was co-purified primarily with trans-3-indoleacrylic acid (manuscript in preparation). The origin of milbemycin oxime in *D. innoxia* extracts is unknown. Antibiotic production by endophytes has been widely observed (Martinez-Klimova et al., 2017), and milbemycin has been reported to be produced by the endophytic fungus *Penicillium citrinum* in the Indian medicinal plant, *Azadirachta indica* (Kumari et al., 2021). Extensive additional studies would be required to determine if milbemycin oxime could have been produced in sufficient amounts by an endophytic microbe with the required biosynthetic gene cluster; by the *D. innoxia* plant (if its genome includes the required biosynthetic gene cluster); or as a contaminant on the leaf surface before collection or during drying.

## Data Availability

The raw data supporting the conclusions of this article will be made available by the authors, without undue reservation.
