# Supplementary material for: Identification of Datura innoxia as a potential source of antimycobacterial components
Source: Front Microbiol. 2025 Jul 1;16:1553282. doi: 10.3389/fmicb.2025.1553282 (PMC12259666; doi:10.3389/fmicb.2025.1553282)
Supplement: Supplementary file 1 [file Data_Sheet_1.docx]

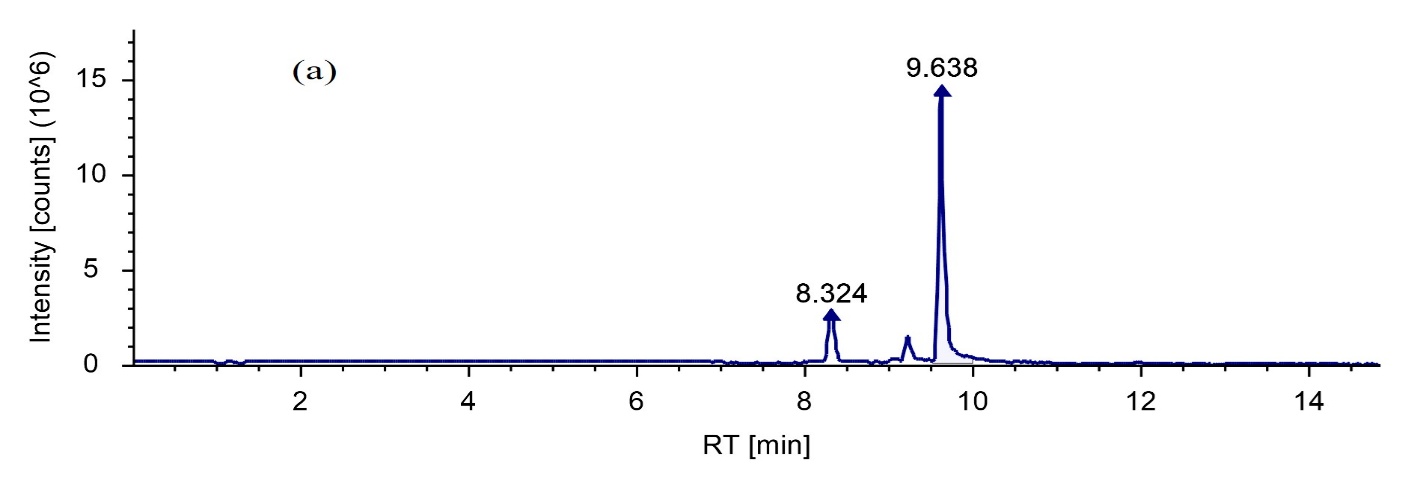

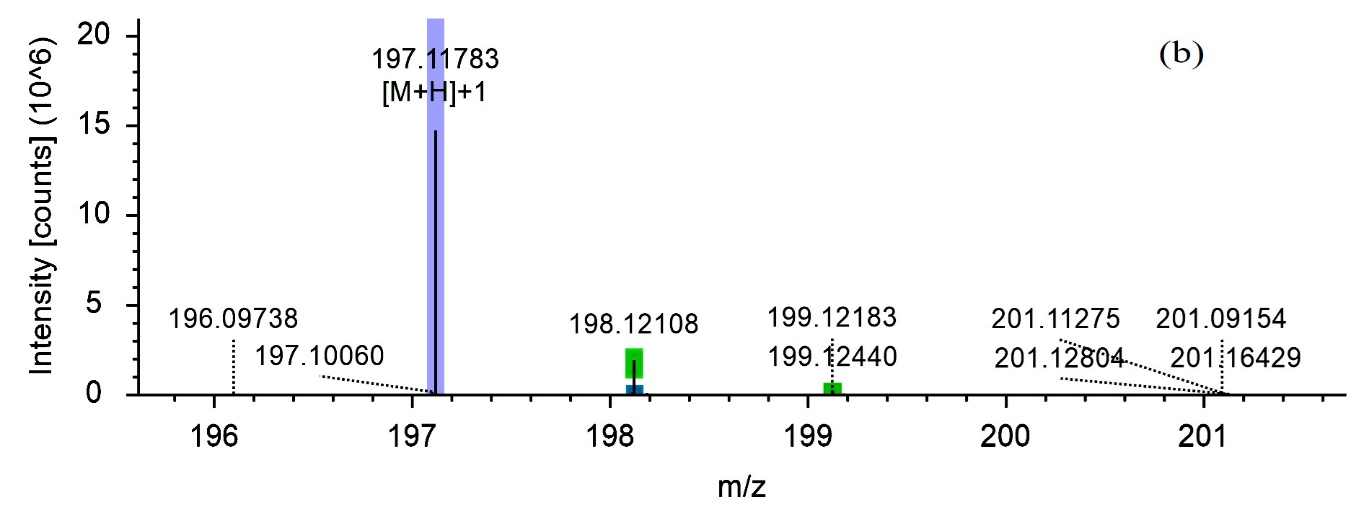


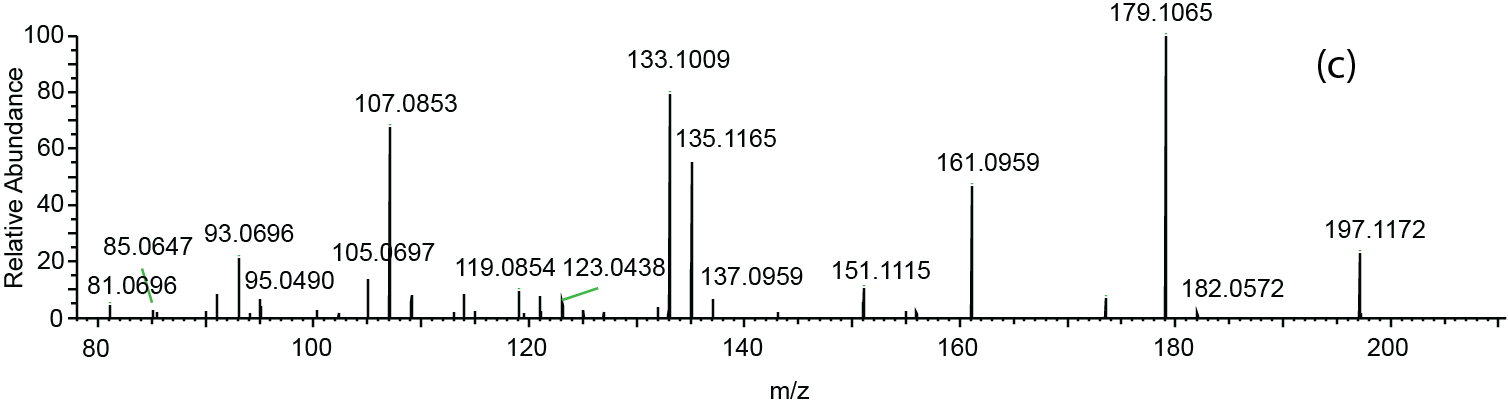


**Supplementary Figure 1:** LC-ESI-MS/MS chromatograms of fraction RC08 in positive ion mode used for the detection of Loliolide. (a) Extracted ion chromatogram, (b) high resolution mass spectrum (MS1), (c) fragmentation mass spectrum for the mass ion at m/z 197.11783 (MS2). RT = retention time.


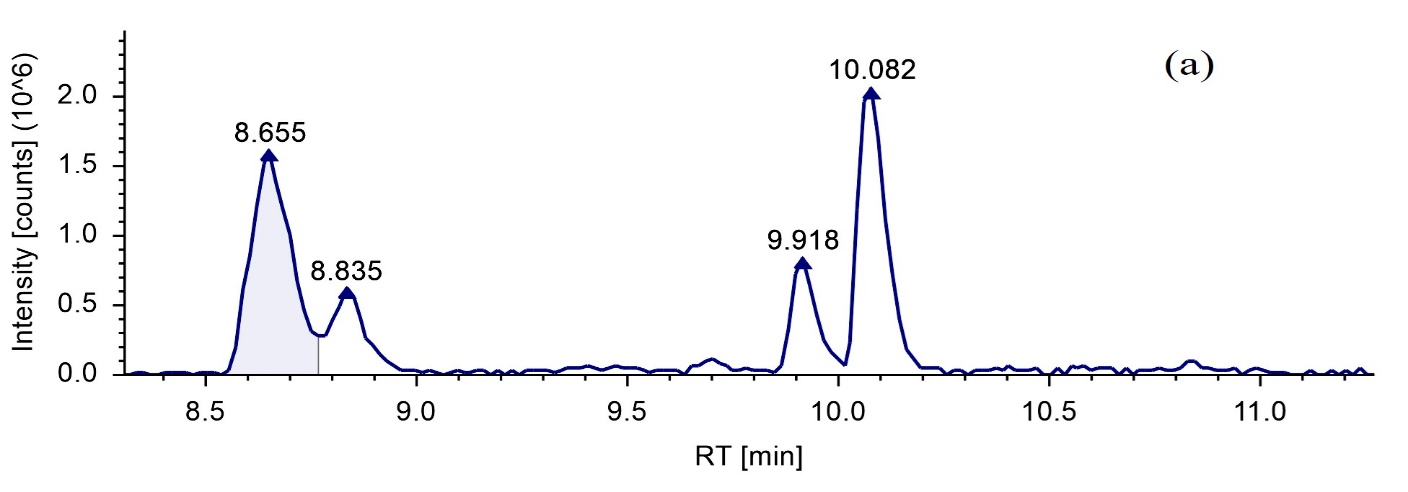

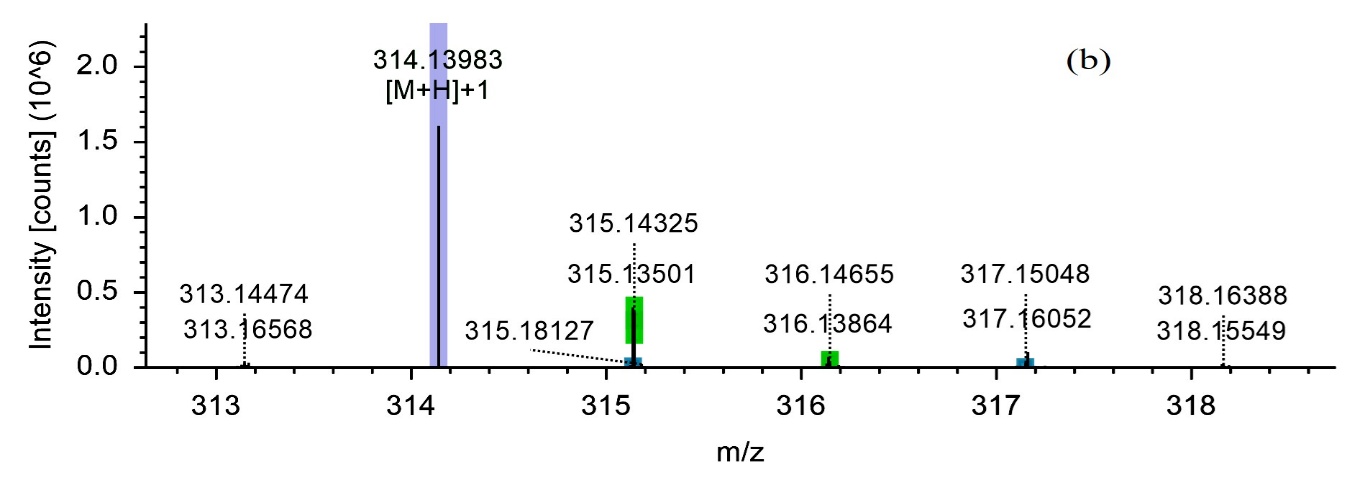

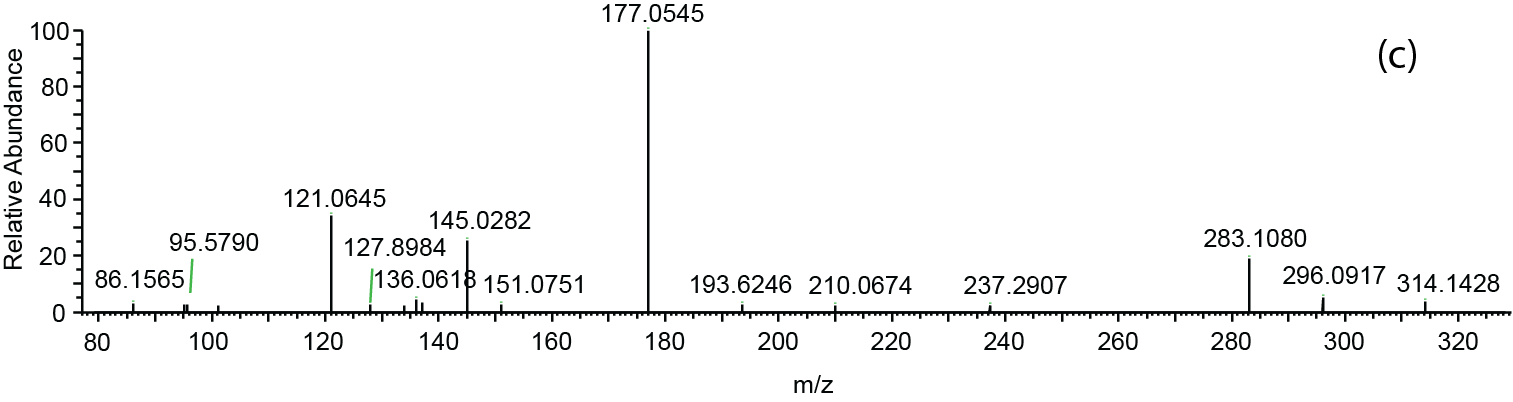


**Supplementary Figure 2:** LC-ESI-MS/MS chromatograms of fraction RC08 in positive ion mode used for the detection of Moupinamide. (a) Extracted ion chromatogram, (b) high resolution mass spectrum (MS1), (c) fragmentation mass spectrum for the mass ion at m/z 314.13983 (MS2). RT = retention time.


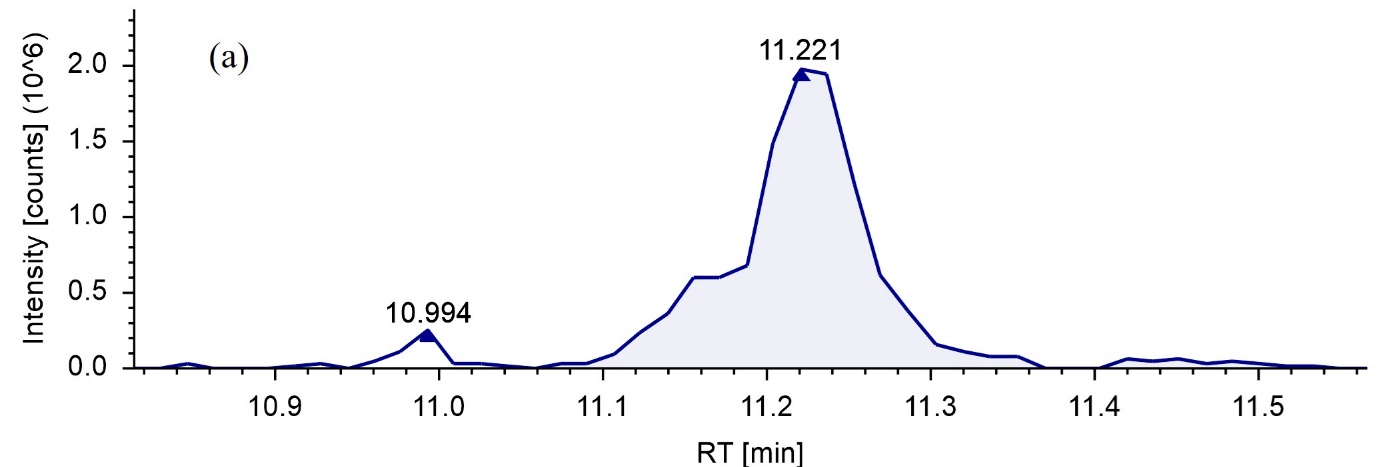

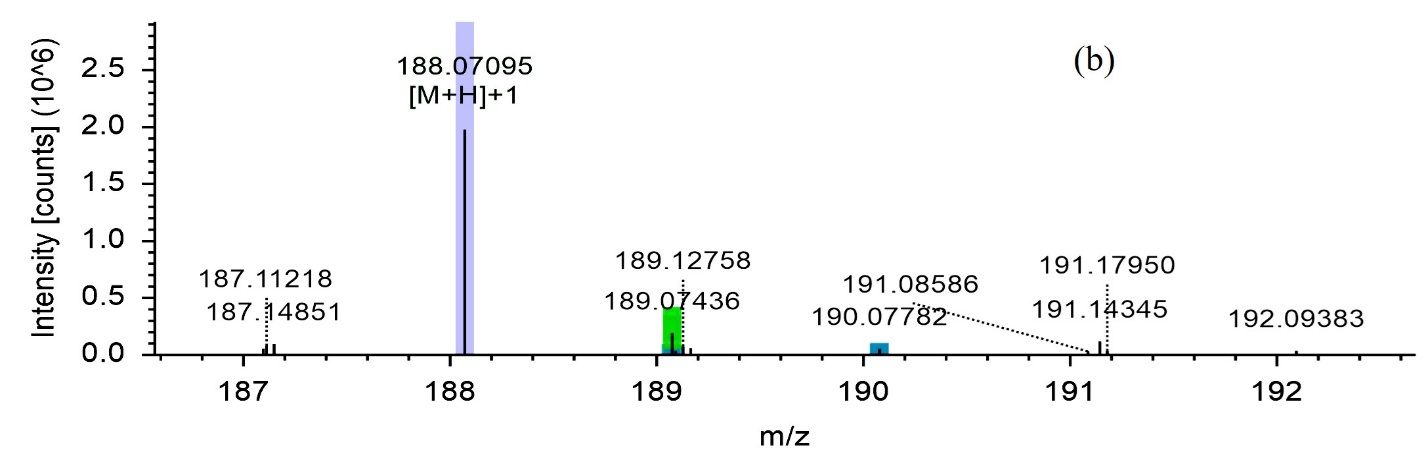

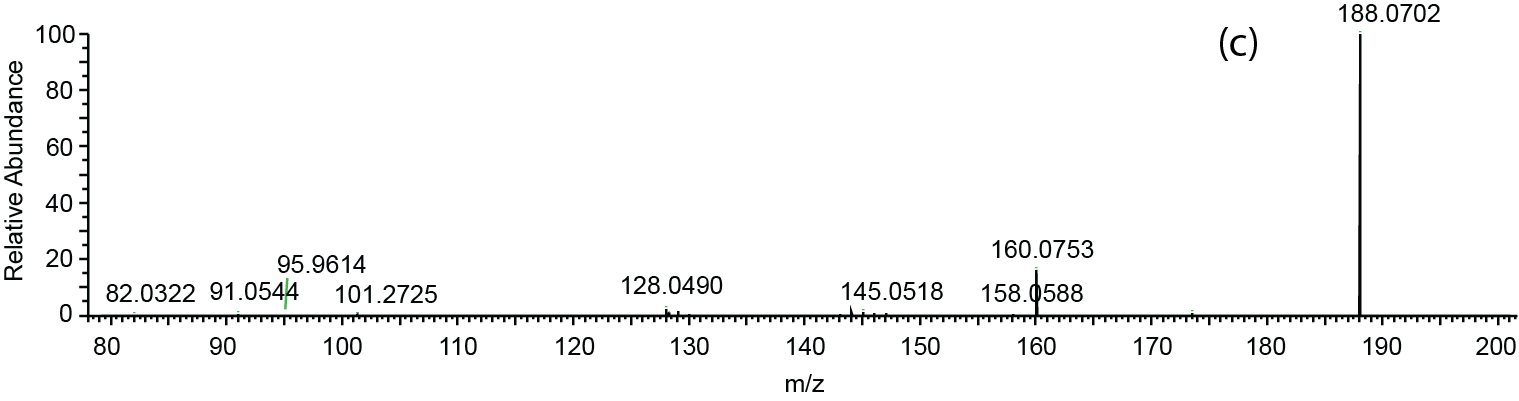


**Supplementary Figure 3**: LC-ESI-MS/MS chromatograms of fraction RC08 in positive ion mode used for the detection of methyl isoquinoline-3-carboxylate. (a) Extracted ion chromatogram, (b) high resolution mass spectrum (MS1), (c) fragmentation mass spectrum for the mass ion at m/z 188.07095 (MS2). RT = retention time.


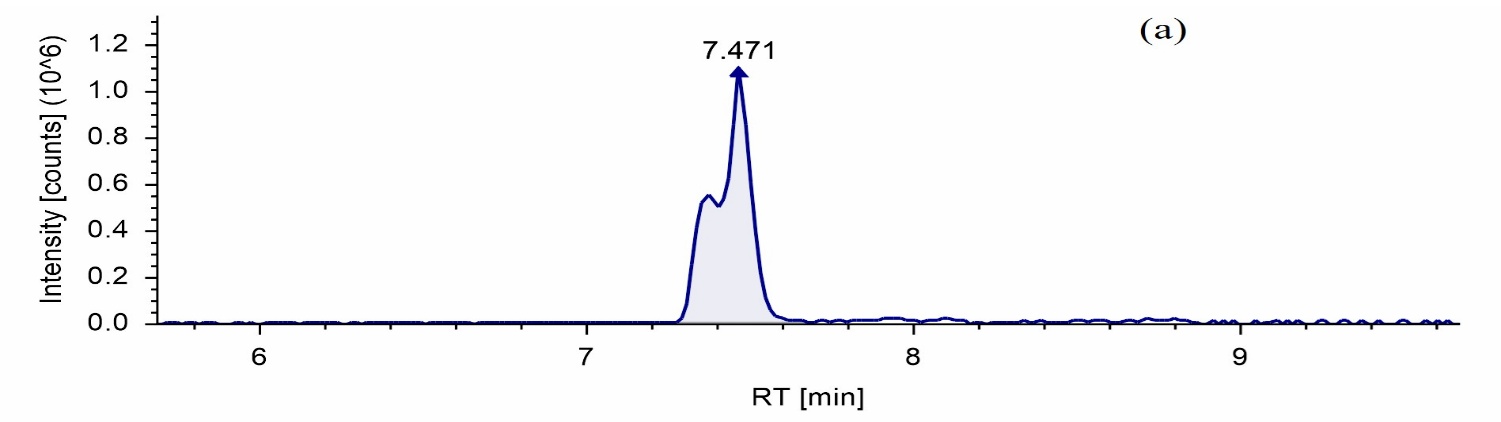

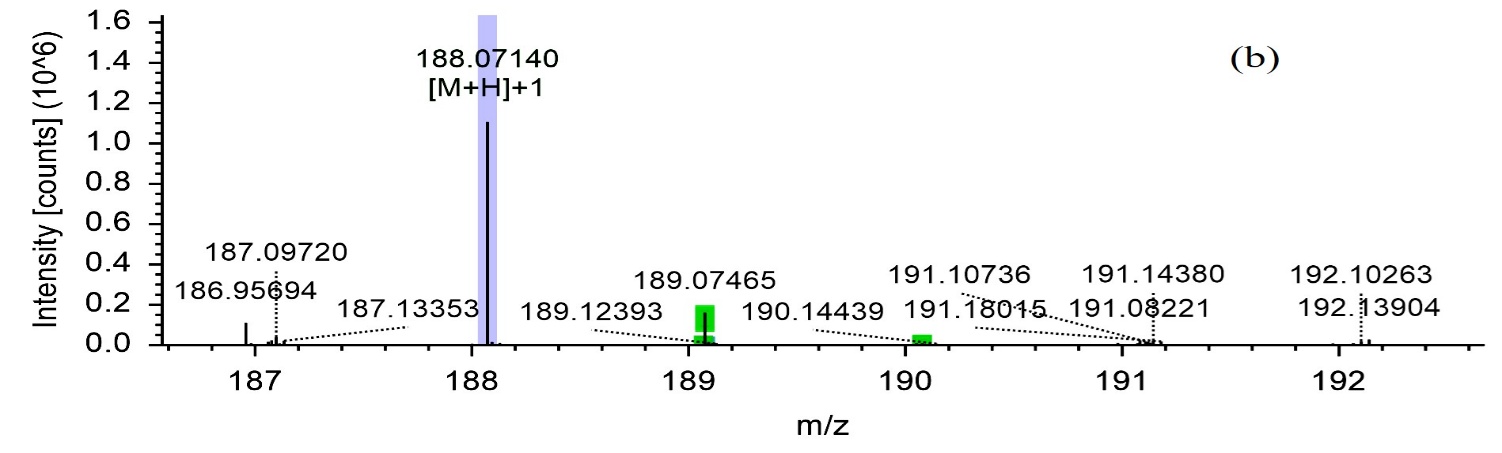

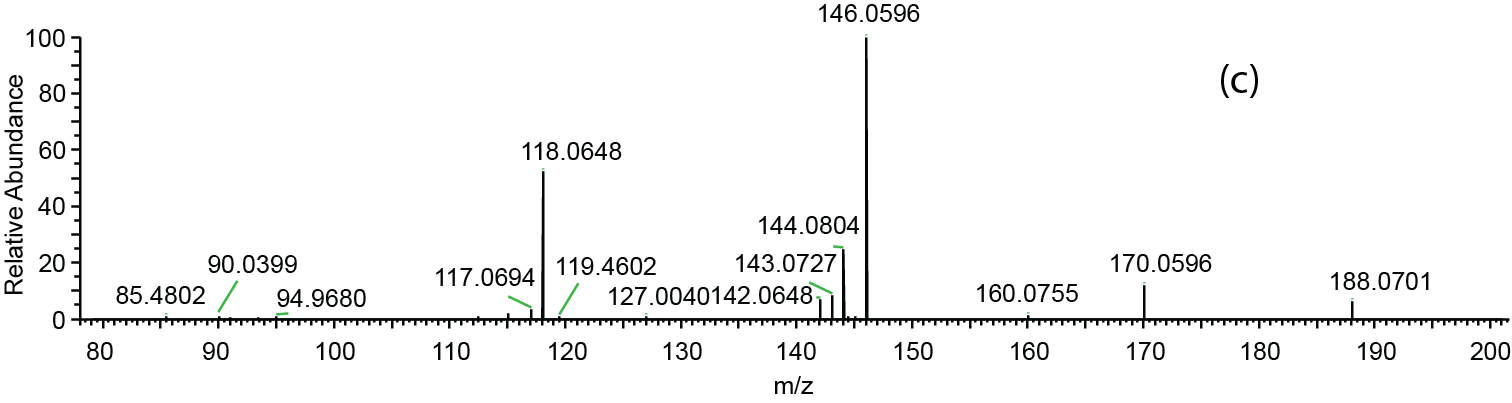


**Supplementary Figure 4:** LC-ESI-MS/MS chromatograms of fraction RC08 in positive ion mode used for the detection of trans-3-Indoleacrylic acid. (a) Extracted ion chromatogram, (b) high resolution mass spectrum (MS1), (c) fragmentation mass spectrum for the mass ion at m/z 188.07140 (MS2). RT = retention time.


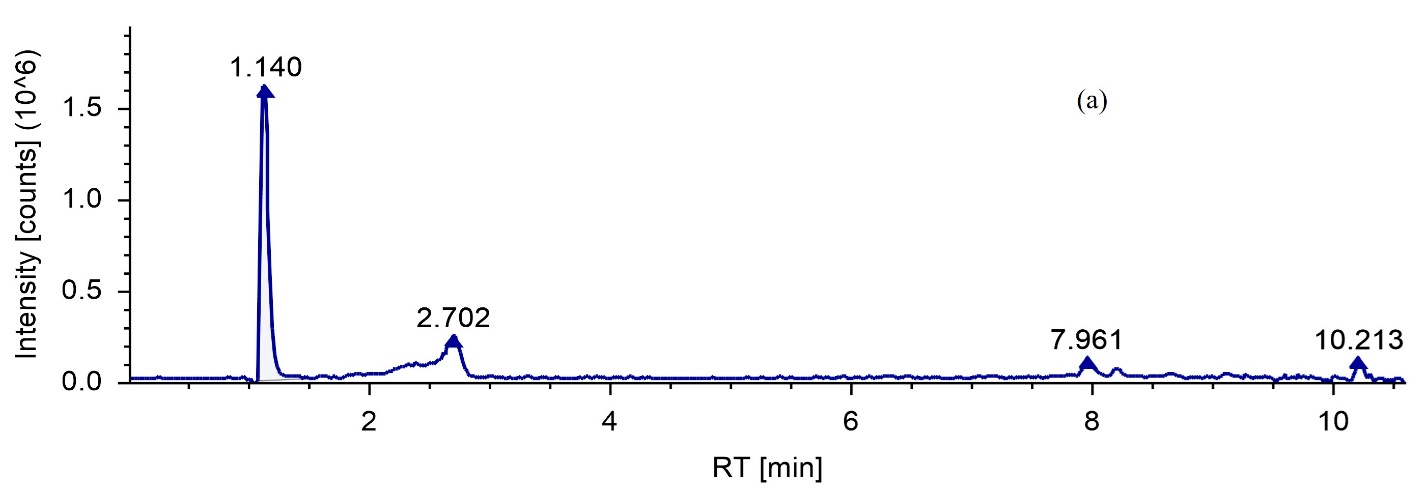

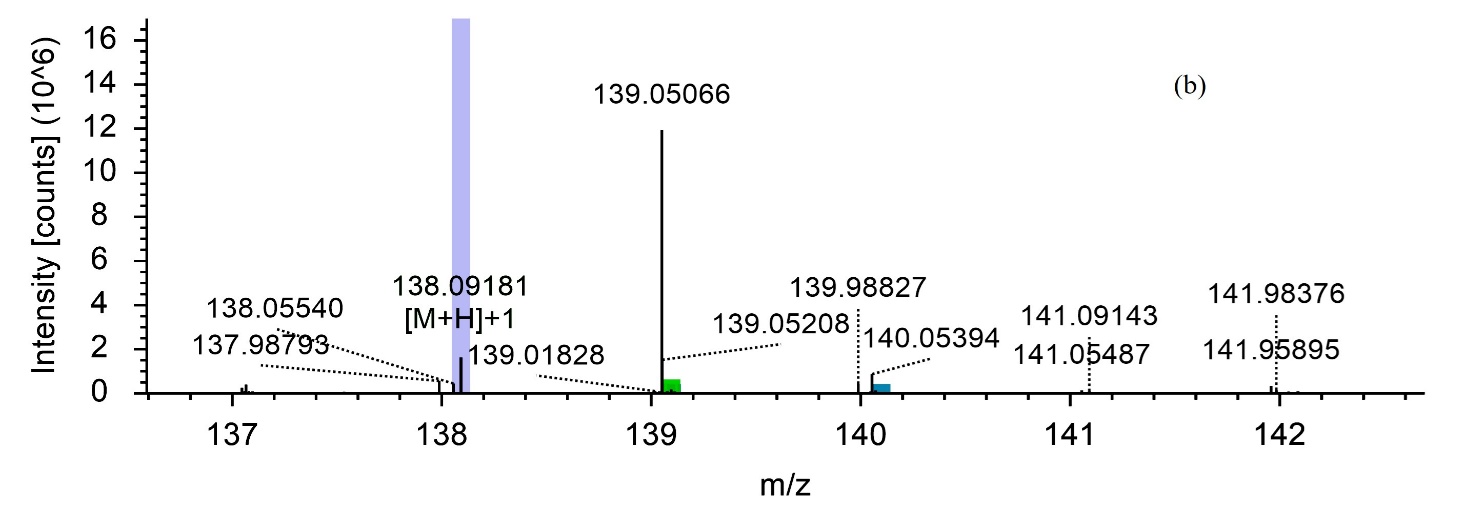


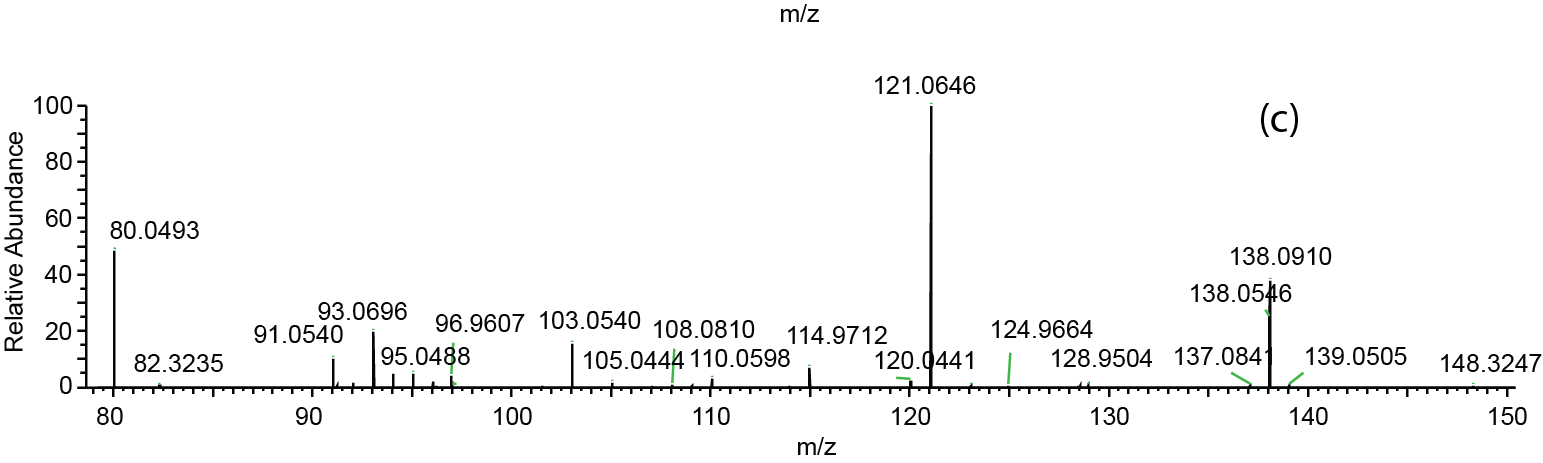


**Supplementary Figure 5:** LC-ESI-MS/MS chromatograms of fraction RC08 in positive ion mode used for the detection of Tyramine. (a) Extracted ion chromatogram, (b) high resolution mass spectrum (MS1), (c) fragmentation mass spectrum for the mass ion at m/z 138.09181 (MS2). RT = retention time.


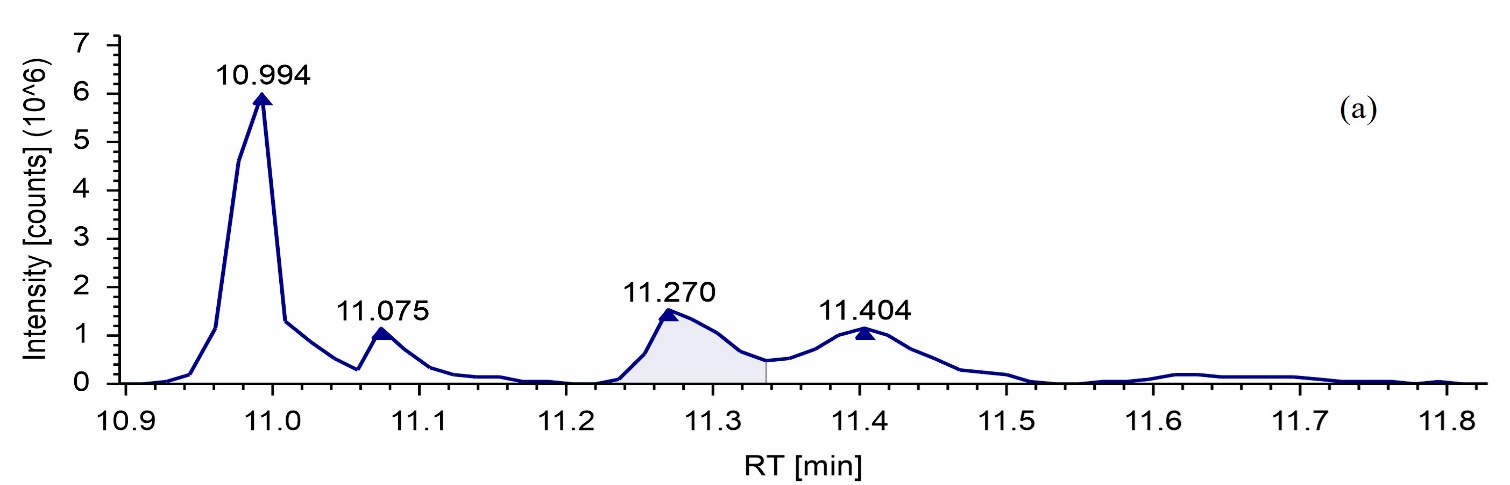

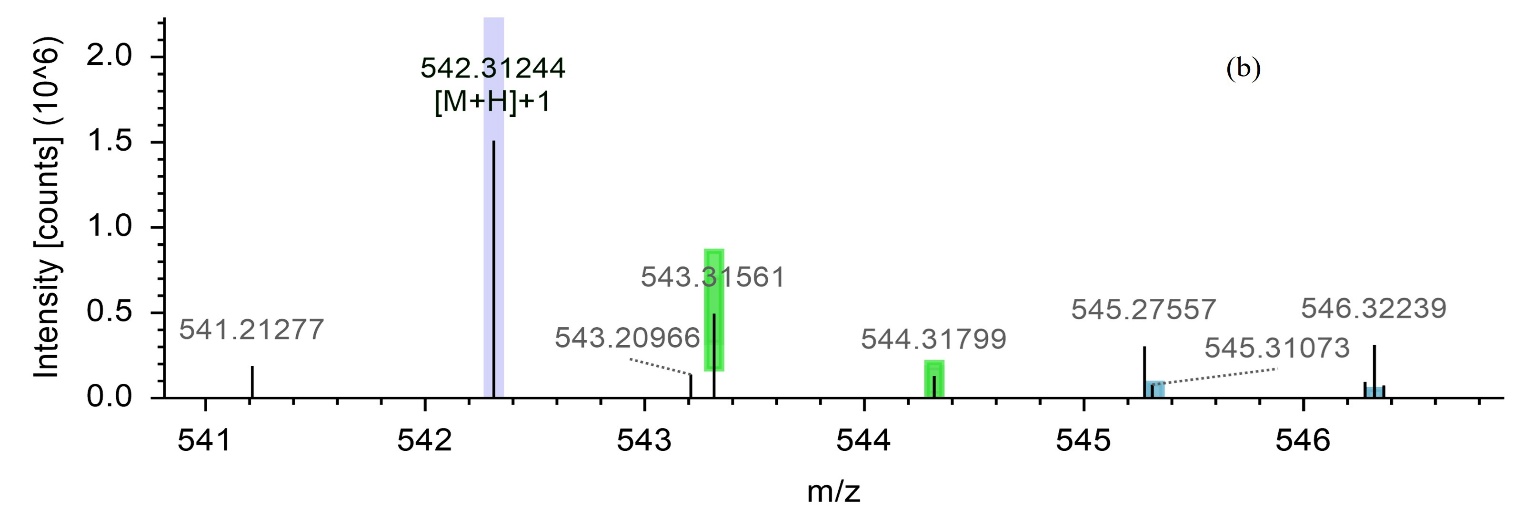

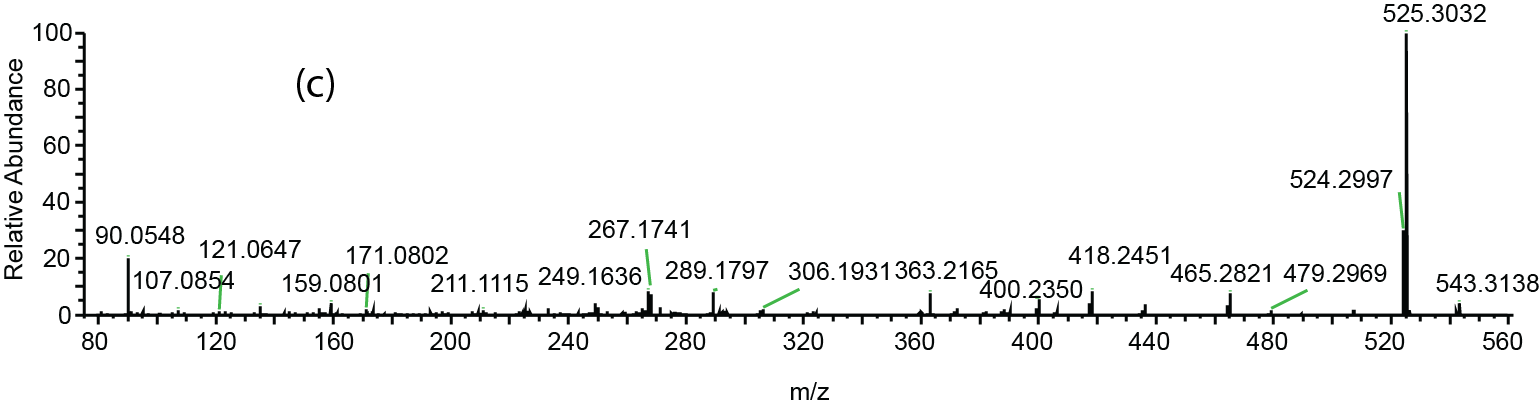


**Supplementary Figure 6:** LC-ESI-MS/MS chromatograms of fraction RC08 in positive ion mode used for the detection of Milbemycin A3 oxime. (a) Extracted ion chromatogram, (b) high resolution mass spectrum (MS1), (c) fragmentation mass spectrum for the mass ion at m/z 542.31244 (MS2). RT = retention time.


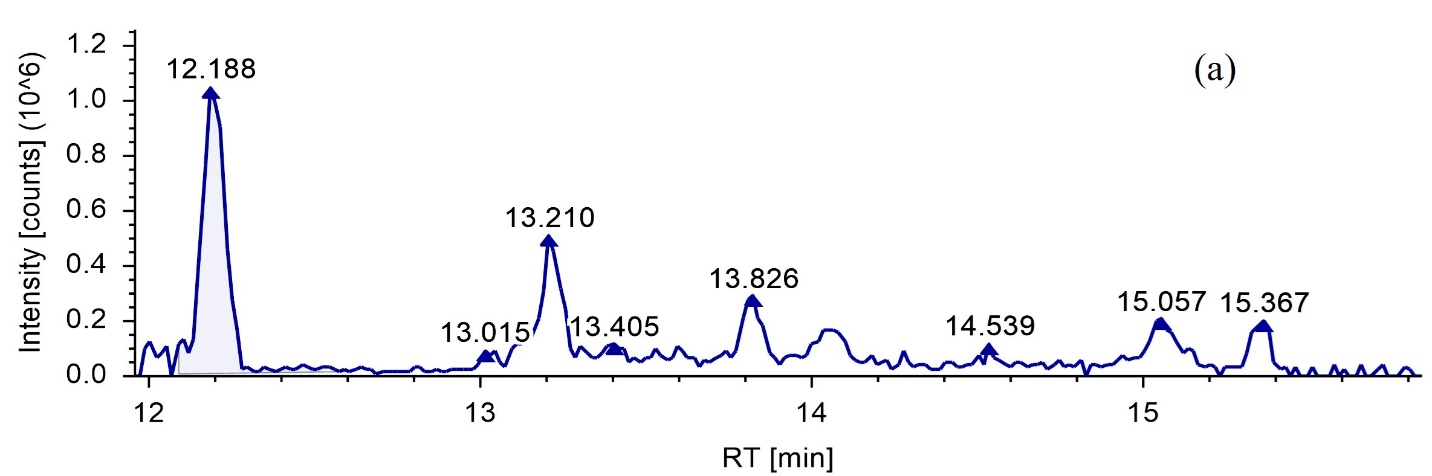

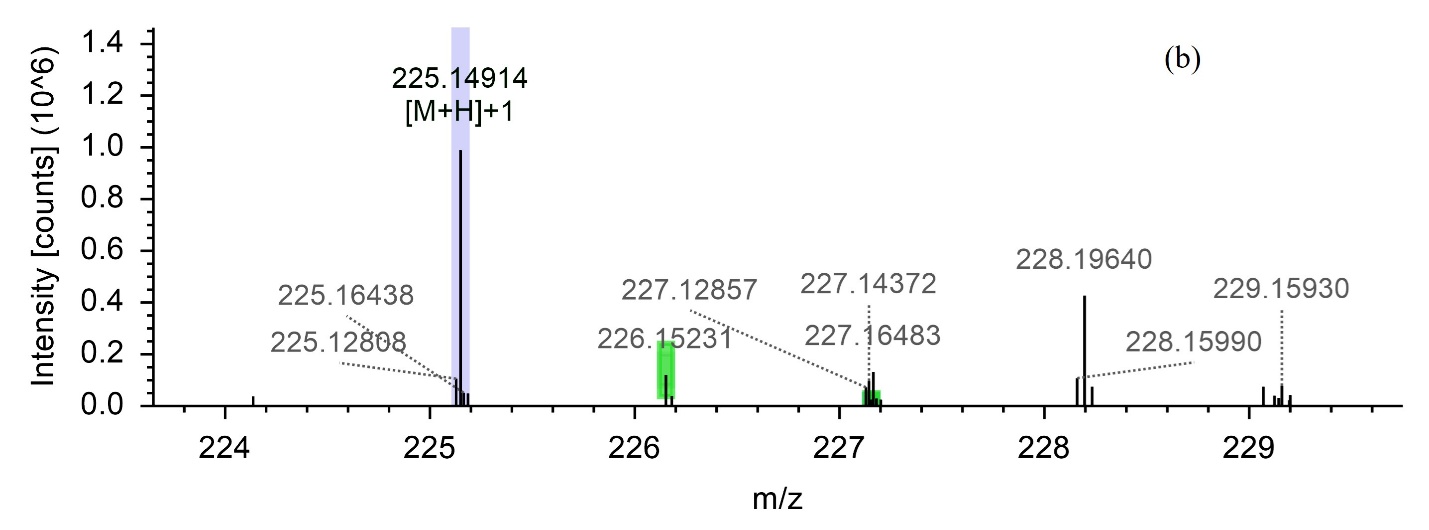


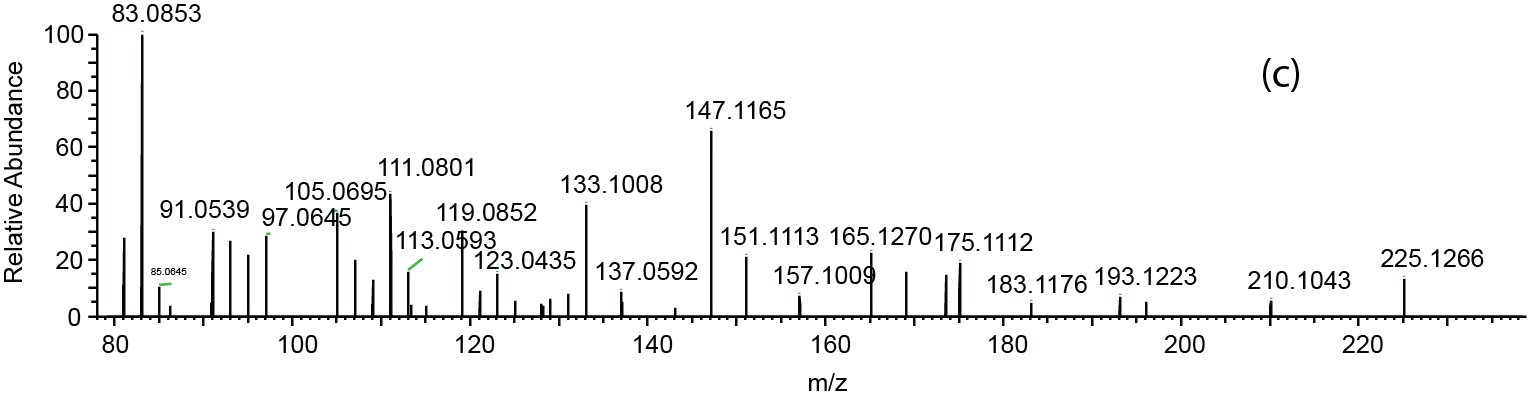


**Supplementary Figure 7**: LC-ESI-MS/MS chromatograms of fraction RC08 in positive ion mode used for the detection of Methyl jasmonate. (a) Extracted ion chromatogram, (b) high resolution mass spectrum (MS1), (c) fragmentation mass spectrum for the mass ion at m/z 225.14914 (MS2). RT = retention time.


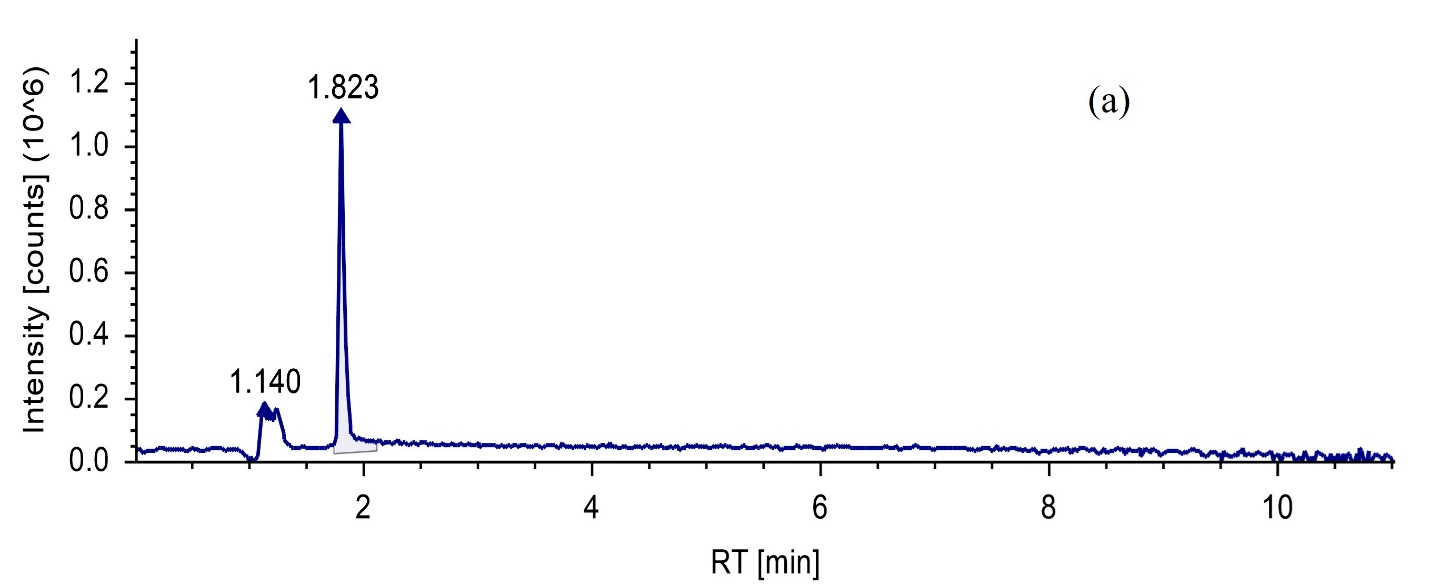

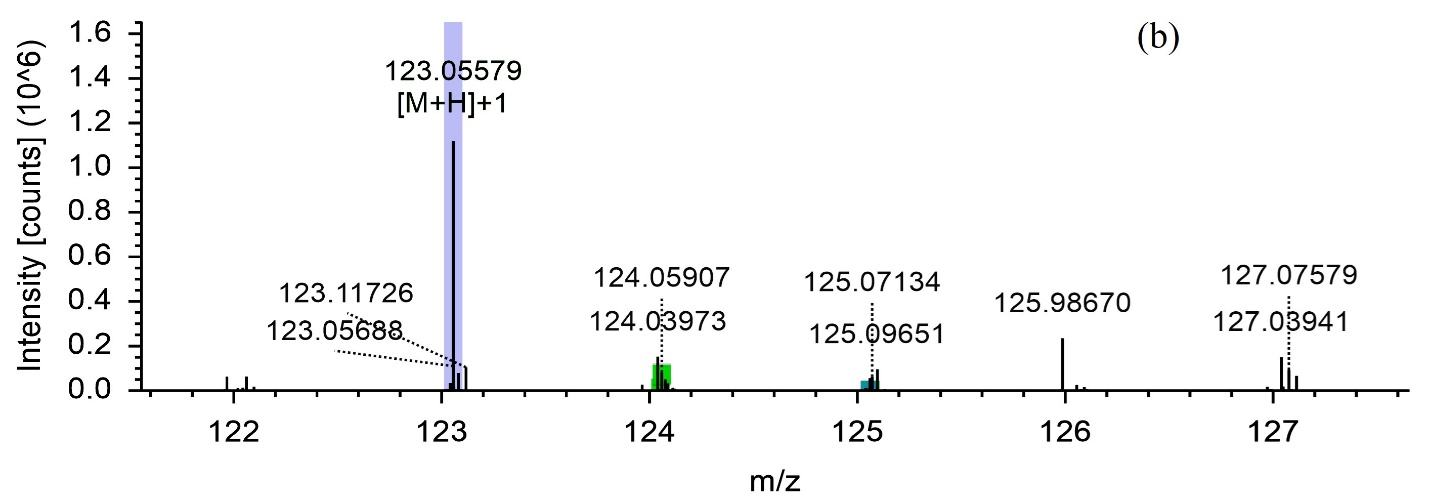

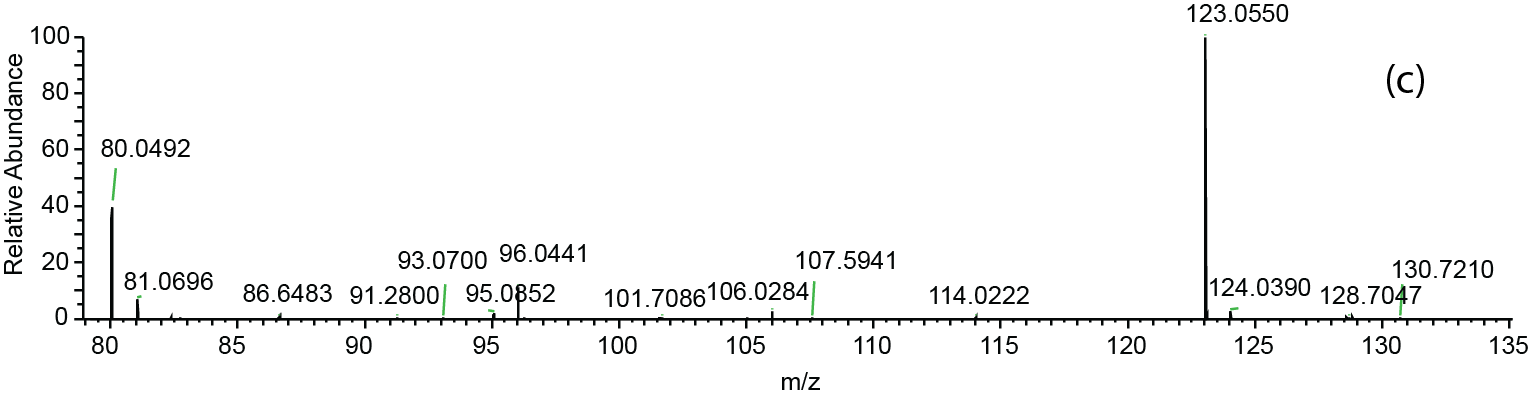


**Supplementary Figure 8**: LC-ESI-MS/MS chromatograms of fraction RC08 in positive ion mode used for the detection of Nicotinamide. (a) Extracted ion chromatogram, (b) high resolution mass spectrum (MS1), (c) fragmentation mass spectrum for the mass ion at m/z 123.05579 (MS2). RT = retention time.


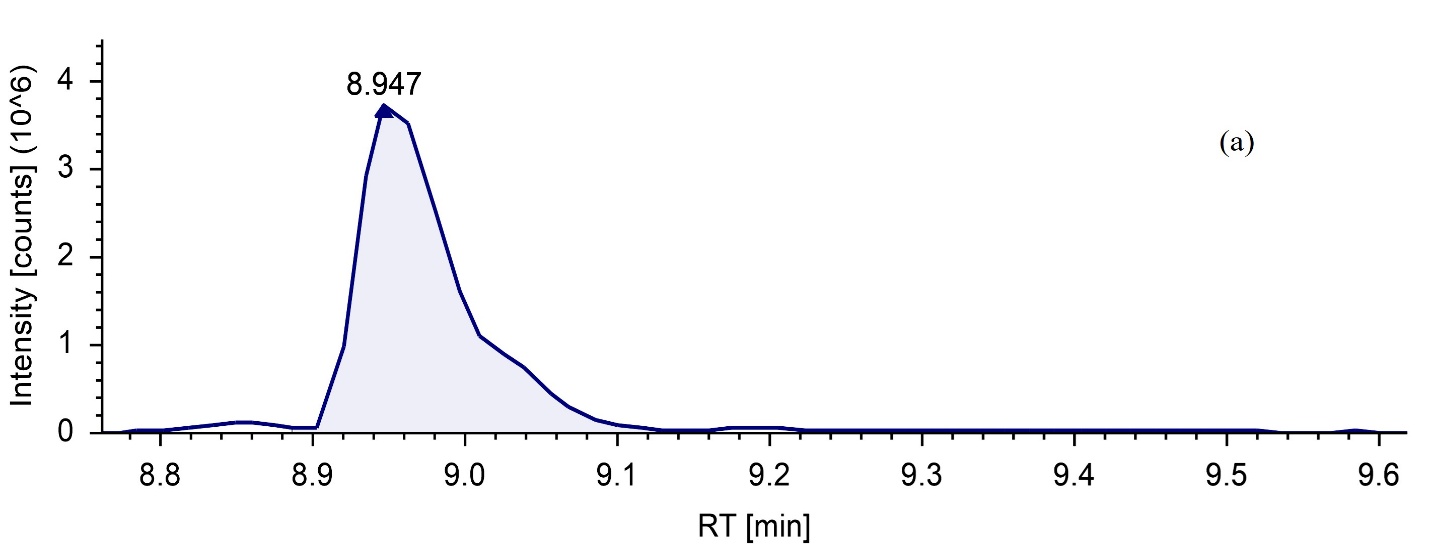

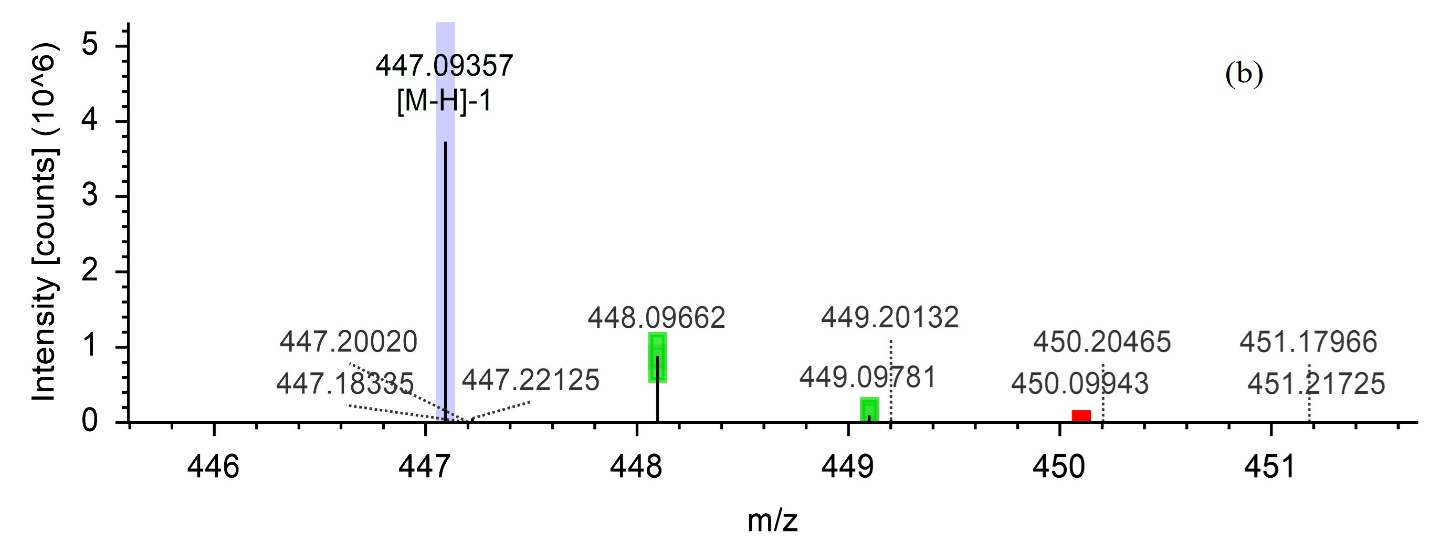

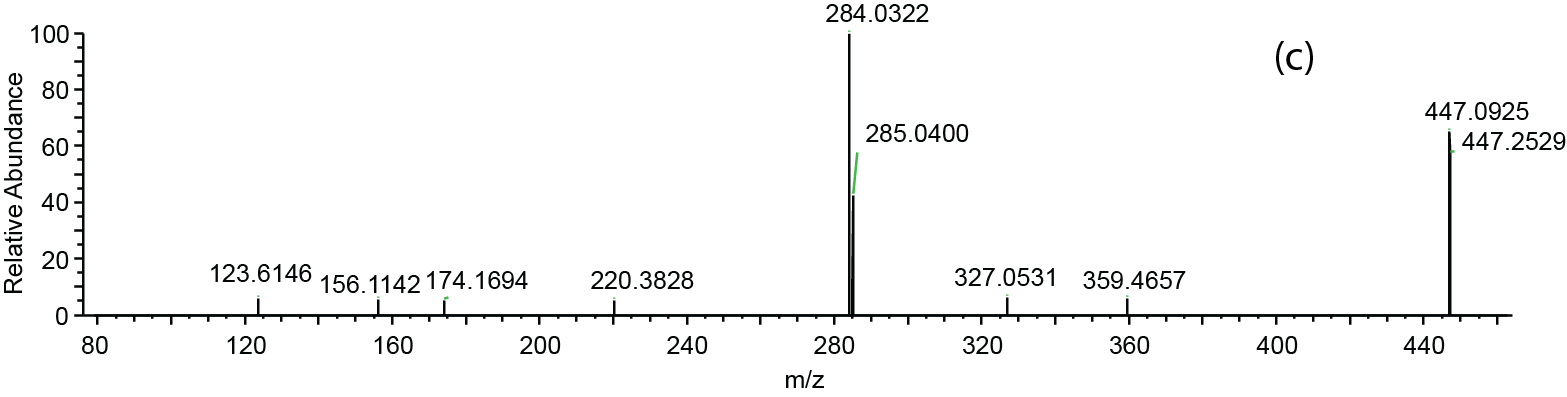


**Supplementary Figure 9:** LC-ESI-MS/MS chromatograms of fraction RC08 in negative ion mode for the detection of Trifolin. (a) Extracted ion chromatogram, (b) high resolution mass spectrum (MS1), (c) fragmentation mass spectrum for the mass ion at m/z 447.09357 (MS2). RT = retention time.


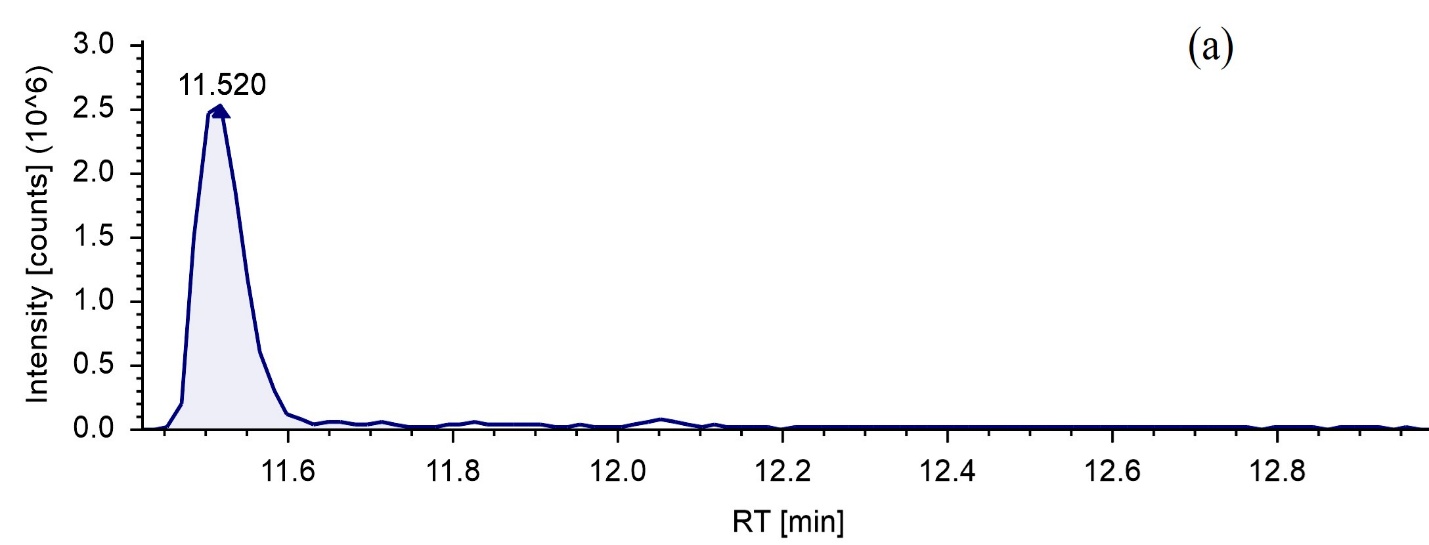

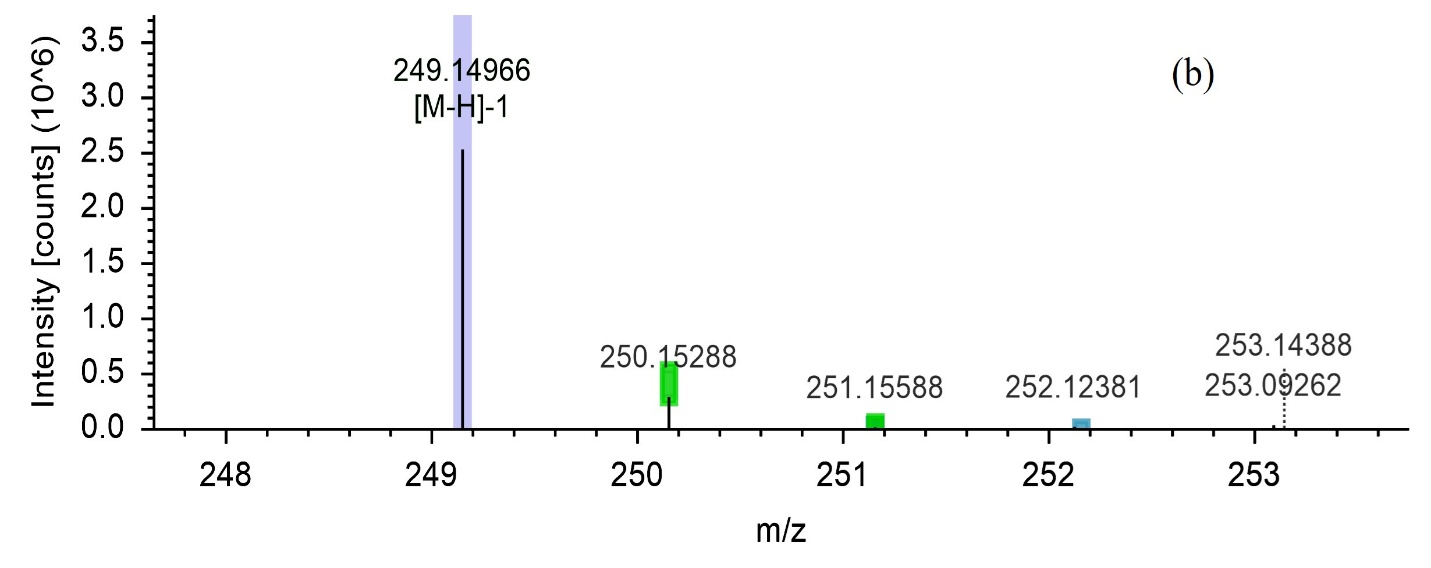

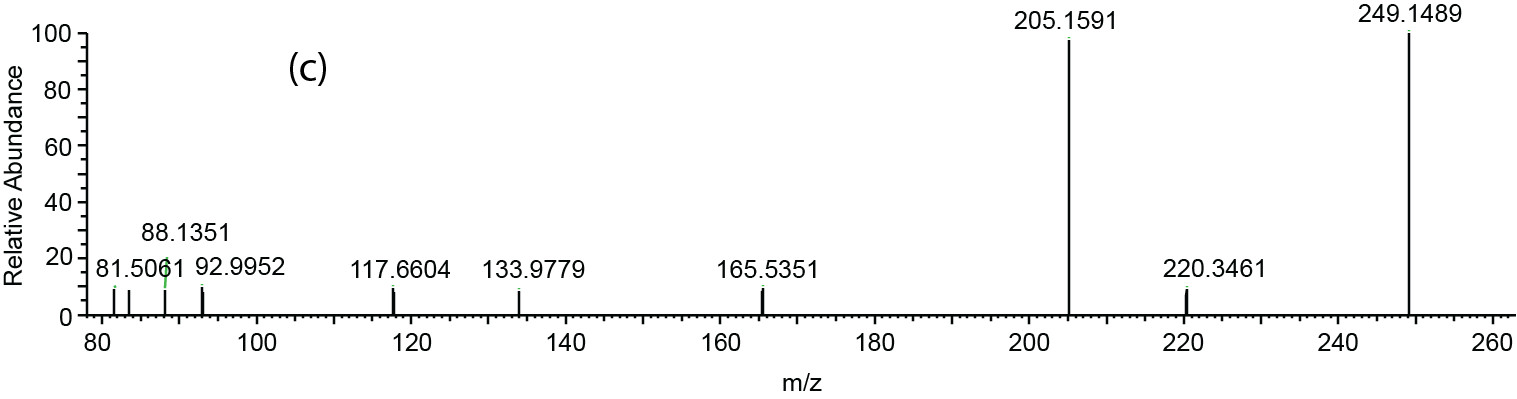


**Supplementary Figure 10**: LC-ESI-MS/MS chromatograms of fraction RC08 in negative ion mode for the detection of 2-[(1S,2S,4aR,8aS)-1-hydroxy-4a-methyl-8-methylidene-decahydronaphthalen-2-yl]prop-2-enoic acid. (a) Extracted ion chromatogram, (b) high resolution mass spectrum (MS1), (c) fragmentation mass spectrum for the mass ion at m/z 249.14966 (MS2). RT = retention time.


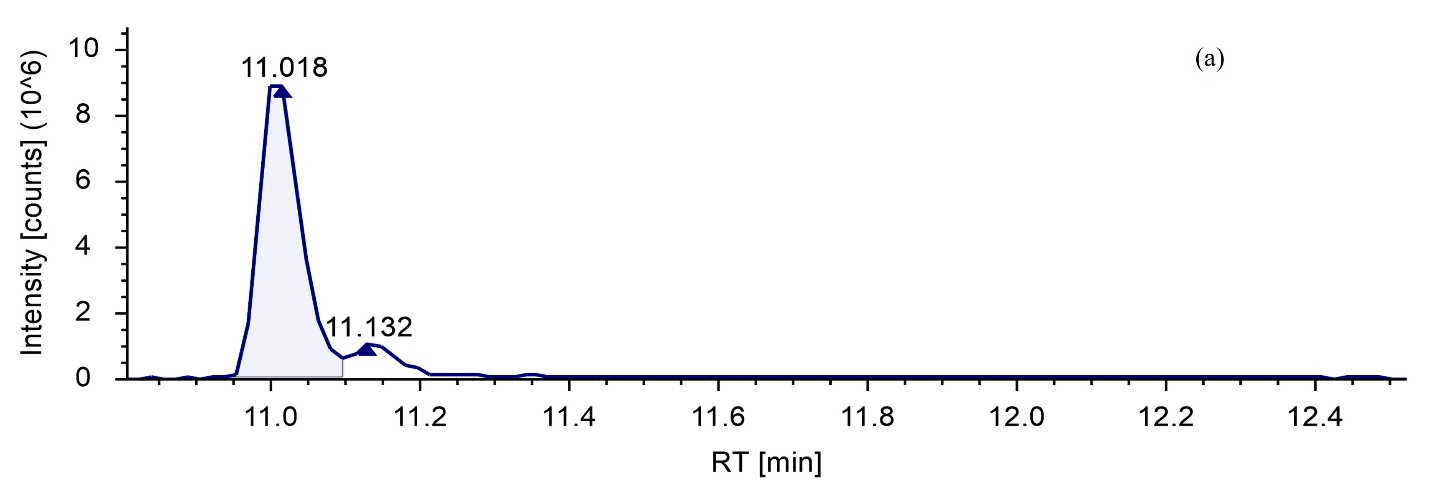

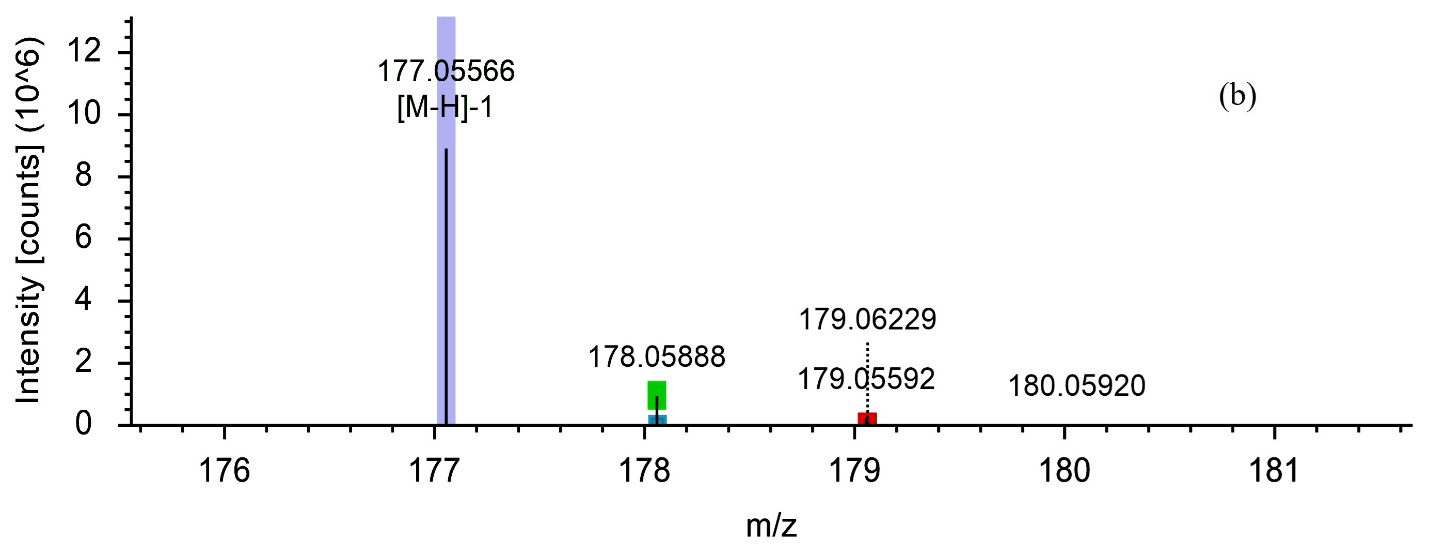

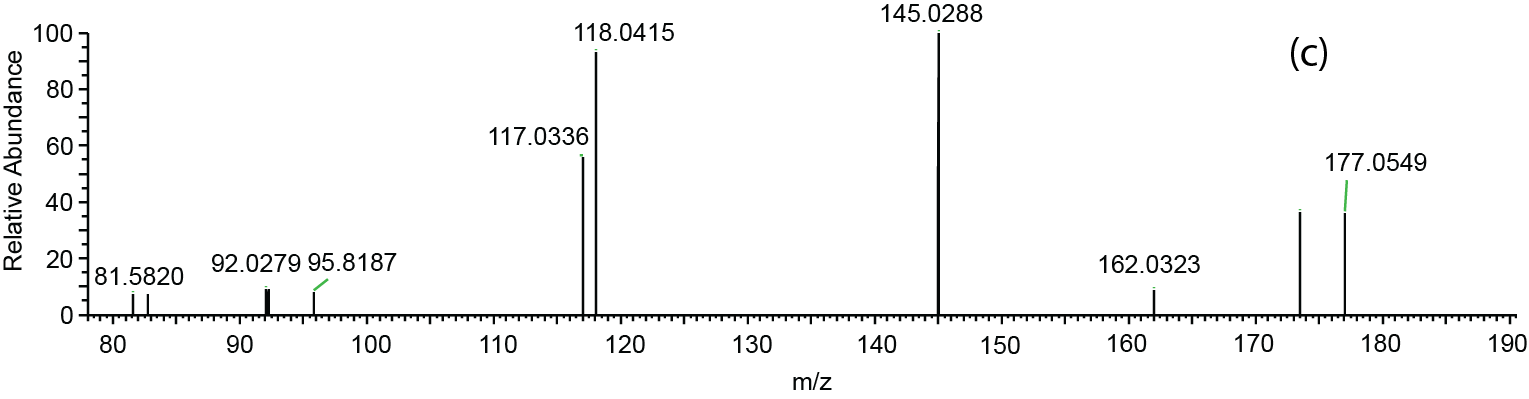


**Supplementary Figure 11:** LC-ESI-MS/MS chromatograms of fraction RC08 in negative ion mode for the detection of Methyl 4-hydroxycinnamate. (a) Extracted ion chromatogram, (b) high resolution mass spectrum (MS1), (c) fragmentation mass spectrum for the mass ion at m/z 177.05566 (MS2). RT = retention time.


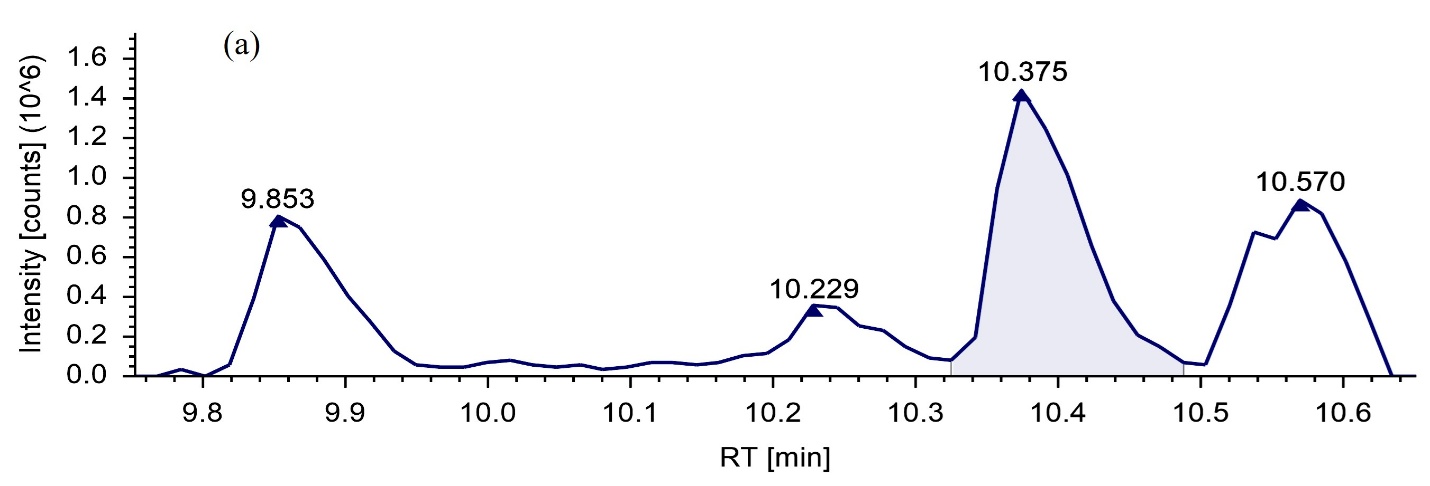

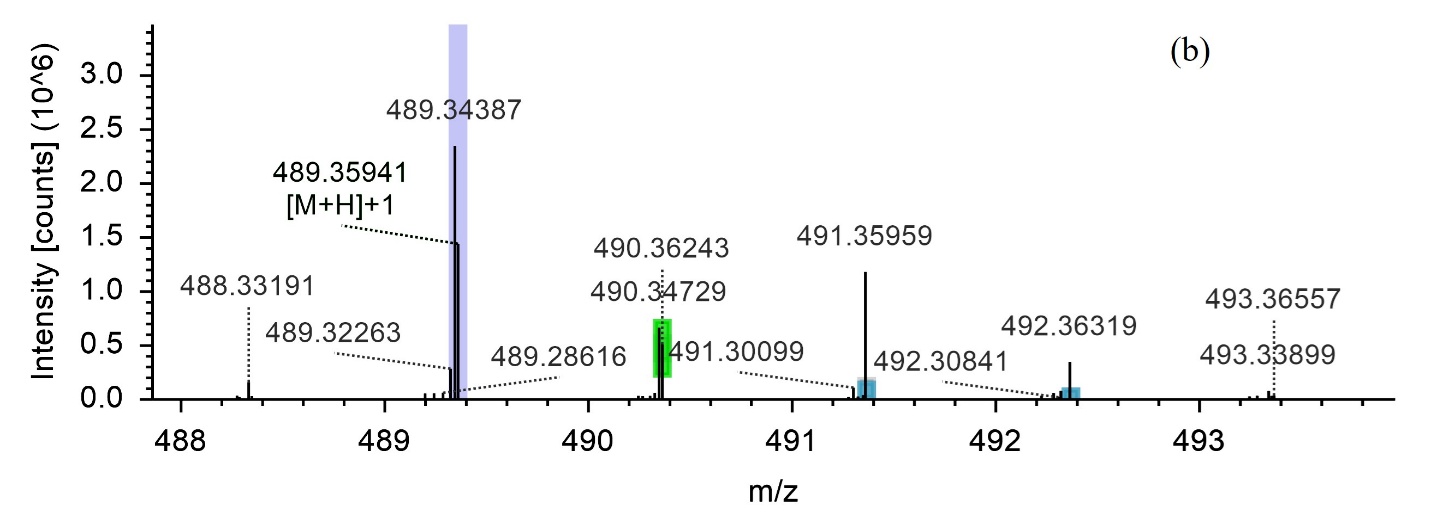

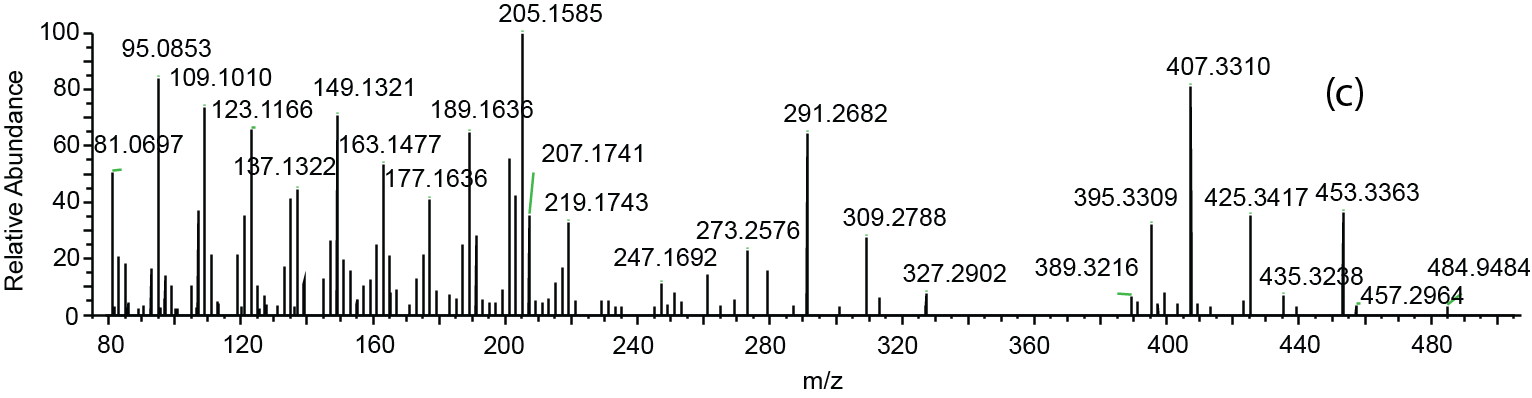


**Supplementary Figure 12:** LC-ESI-MS/MS chromatograms of fraction RC08 in positive ion mode used for the detection of (3β,5ξ,9ξ)-3,6,19-Trihydroxyurs-12-en-28-oic acid. (a) Extracted ion chromatogram, (b) high resolution mass spectrum (MS1), (c) fragmentation mass spectrum for the mass ion at m/z 489.34387 (MS2). RT = retention time.


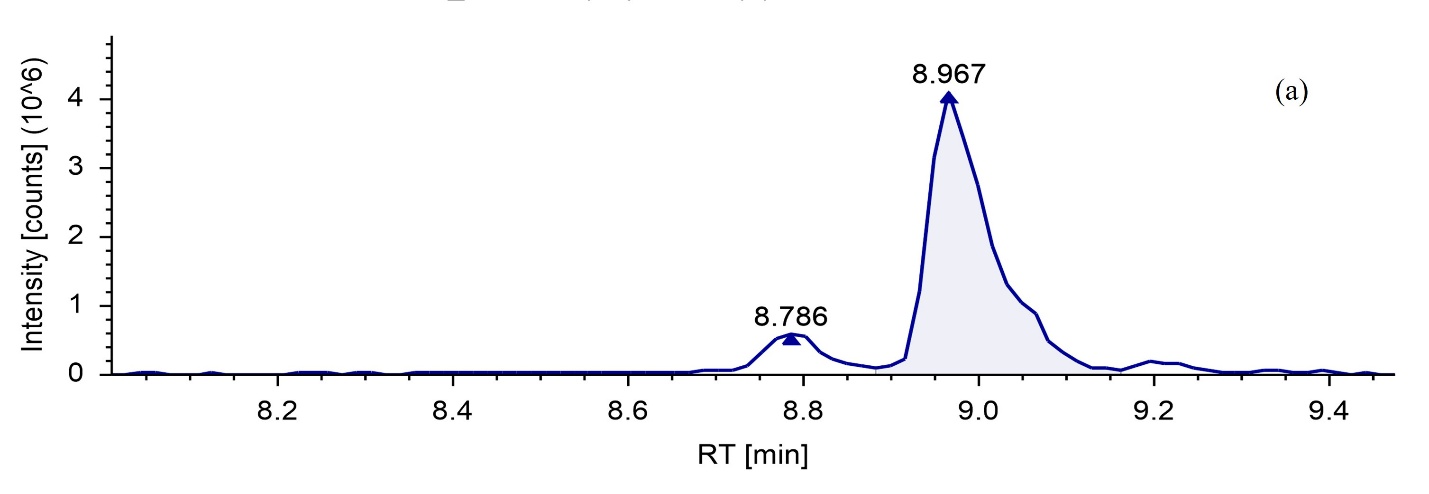

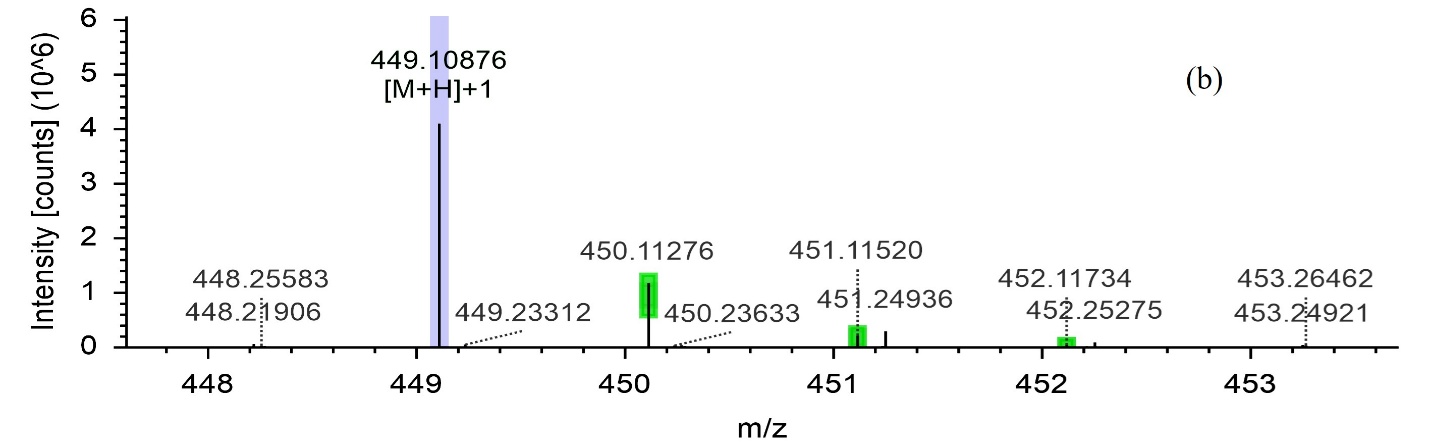

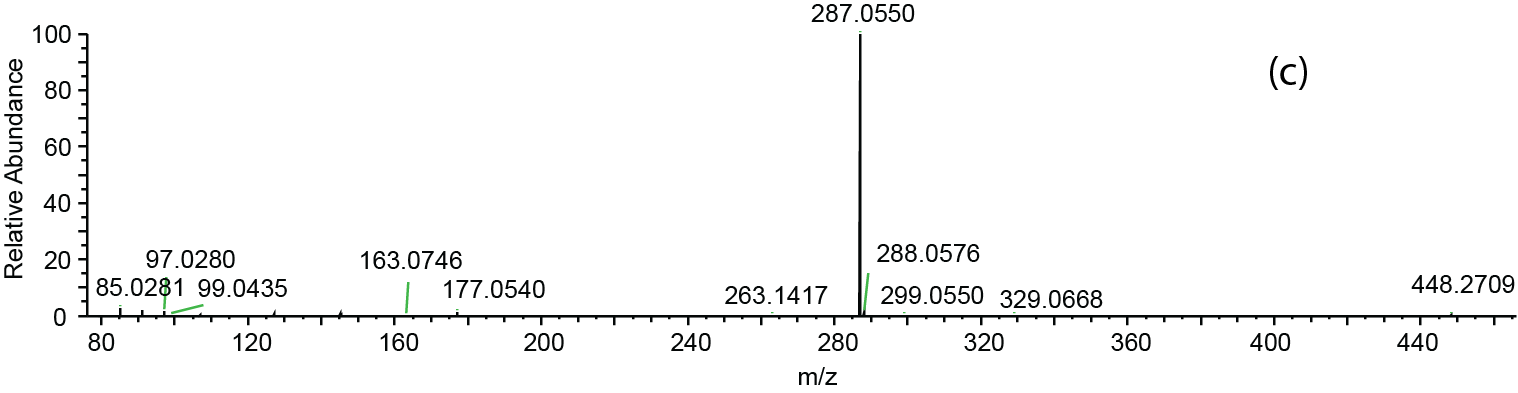


**Supplementary Figure 13:** LC-ESI-MS/MS chromatograms of fraction RC08 in positive ion mode used for the detection of Kuromanin. (a) Extracted ion chromatogram, (b) high resolution mass spectrum (MS1), (c) fragmentation mass spectrum for the mass ion at m/z 499.10876 (MS2). RT = retention time.


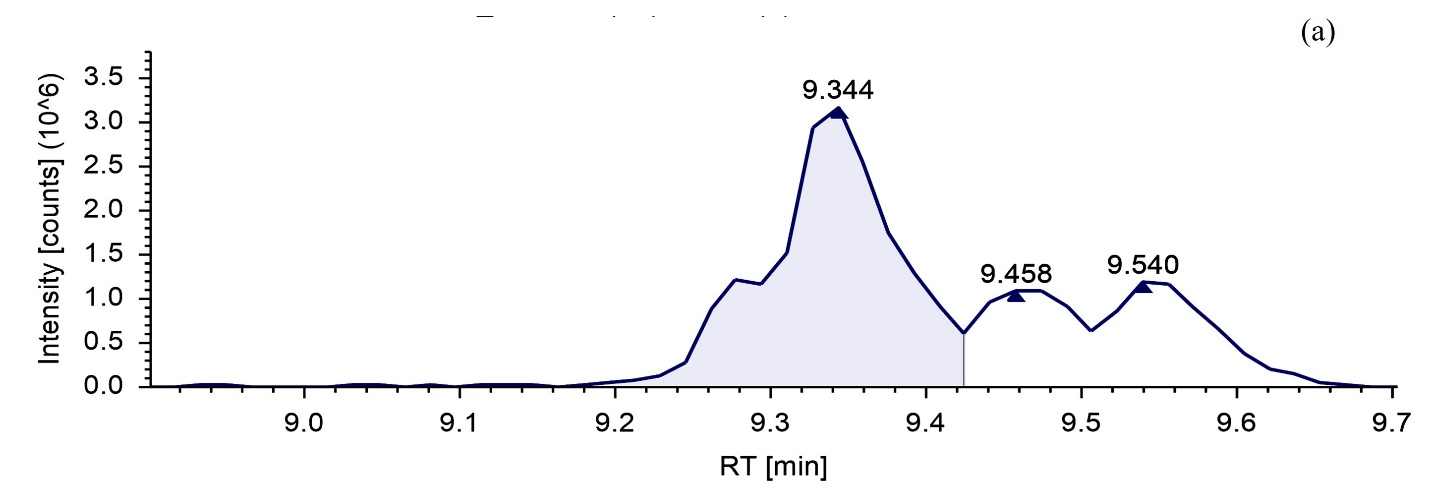

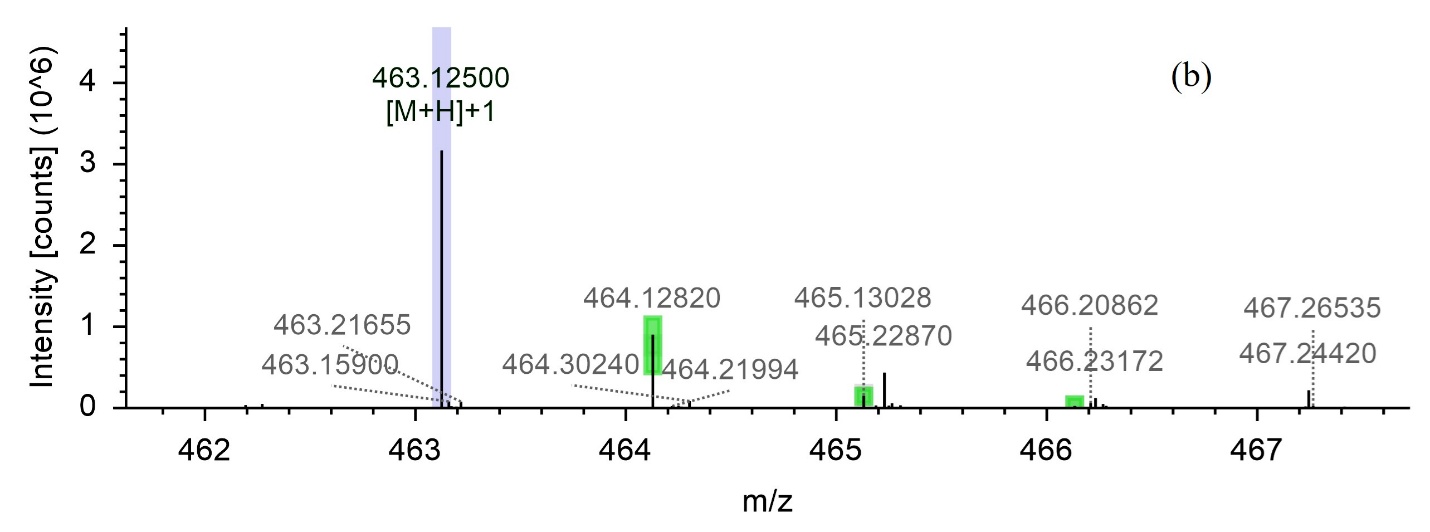

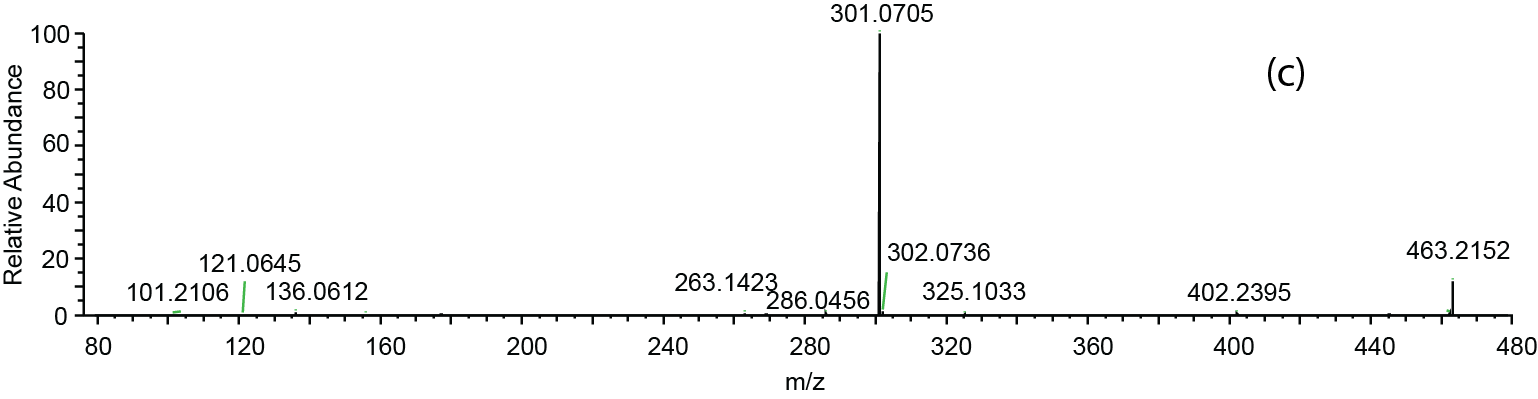


**Supplementary Figure 14**: LC-ESI-MS/MS chromatograms of fraction RC08 in positive ion mode used for the detection of Isoquercitrin. (a) Extracted ion chromatogram, (b) high resolution mass spectrum (MS1), (c) fragmentation mass spectrum for the mass ion at m/z 463.12500 (MS2). RT = retention time.

(a)


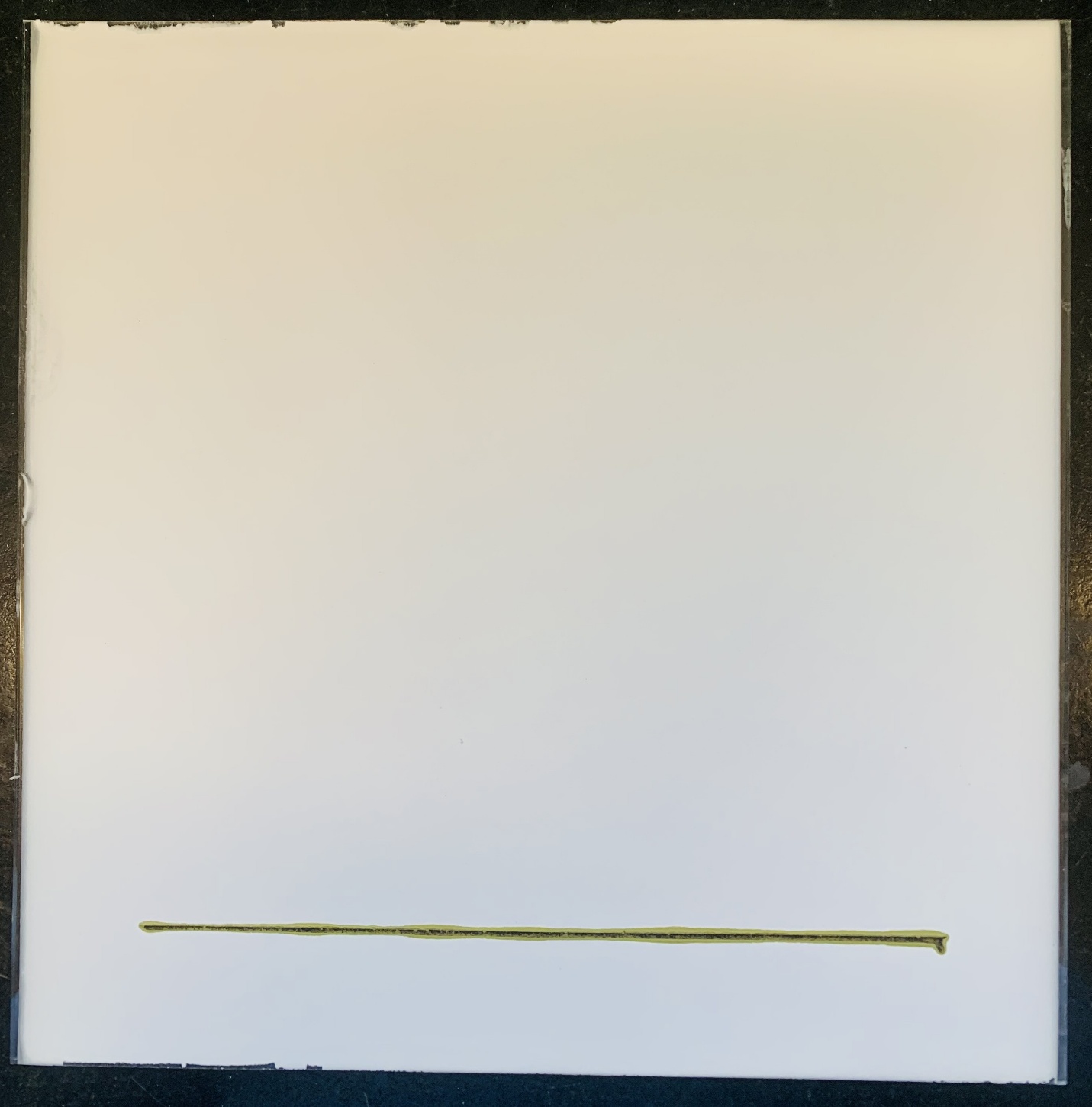


(b)
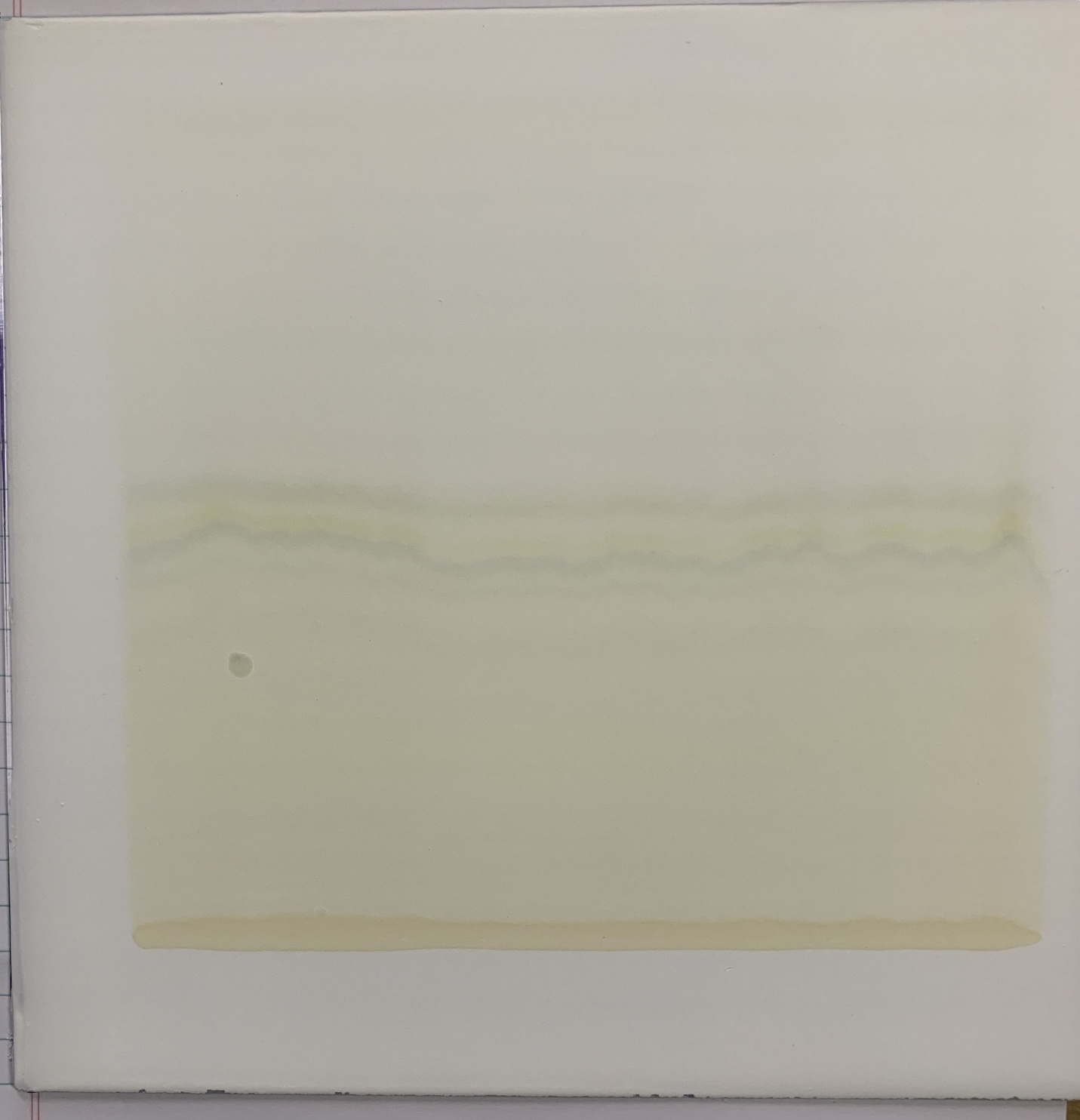


(c)
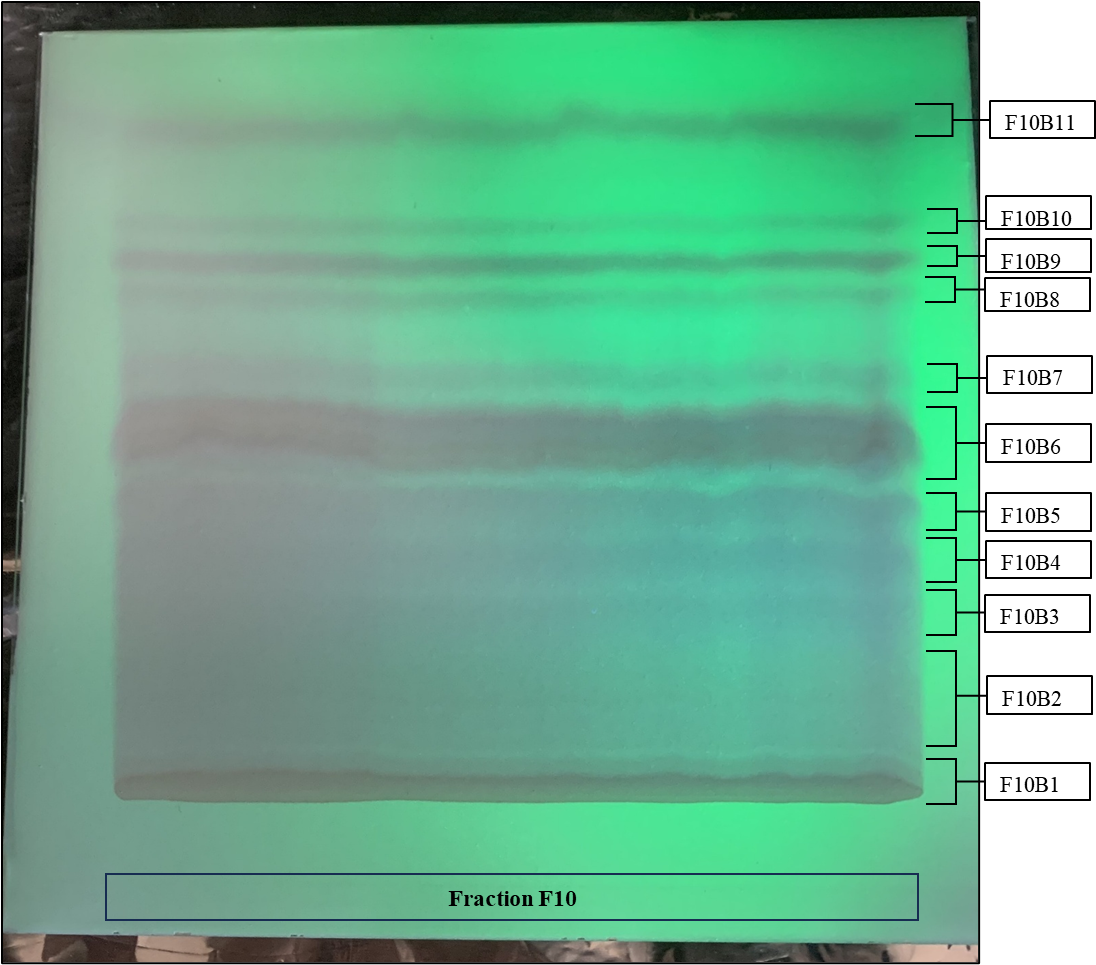


**Supplementary Figure 15: Preparative thin-layer chromatography (PTLC) of fraction F10.** **(a)** Application of plant extract on a preparative thin-layer chromatography (PTLC) plate before development. **(b)** Developed PTLC plate showing the separation of bands from the plant extract using a mobile phase of chloroform (75%) and ethanol (25%). **(c)** Visualization of separated bands under UV light.
